# Distinct spatiotemporal atrophy patterns in corticobasal syndrome are associated with different underlying pathologies

**DOI:** 10.1093/braincomms/fcaf066

**Published:** 2025-02-11

**Authors:** William J Scotton, Cameron Shand, Emily G Todd, Martina Bocchetta, Christopher Kobylecki, David M Cash, Lawren VandeVrede, Hilary W Heuer, Annelies Quaegebeur, Alexandra L Young, Neil Oxtoby, Daniel Alexander, James B Rowe, Huw R Morris, for the PROSPECT Consortium, Bradley F Boeve, Brad C Dickerson, Carmela M Tartaglia, Irene Litvan, Murray Grossman, Alexander Pantelyat, Edward D Huey, David J Irwin, Anne Fagan, Suzanne L Baker, Arthur W Toga, Adam L Boxer, for the 4RTNI Consortium, Edwin Jabbari, Marte Theilmann Jensen, Danielle Lux, Riona Fumi, David P Vaughan, Henry Houlden, Michele T M Hu, Peter N Leigh, Jonathan D Rohrer, Peter A Wijeratne

**Affiliations:** Dementia Research Centre, Department of Neurodegenerative Disease, University College London Queen Square Institute of Neurology, University College London, London WC1N 3AR, UK; Centre for Medical Image Computing, Department of Computer Science, University College London, London WC1V 6LJ, UK; Dementia Research Centre, Department of Neurodegenerative Disease, University College London Queen Square Institute of Neurology, University College London, London WC1N 3AR, UK; Dementia Research Centre, Department of Neurodegenerative Disease, University College London Queen Square Institute of Neurology, University College London, London WC1N 3AR, UK; Centre for Cognitive and Clinical Neuroscience, Division of Psychology, Department of Life Sciences, College of Health, Medicine and Life Sciences, Brunel University, London UB8 3PH, UK; Department of Neurology, Manchester Centre for Clinical Neurosciences, Northern Care Alliance NHS Foundation Trust (Salford Royal Hospital), Salford M6 8HD, UK; Division of Neuroscience, Faculty of Biology, Medicine and Health, School of Biological Sciences, The University of Manchester, Manchester M13 9PT, UK; Dementia Research Centre, Department of Neurodegenerative Disease, University College London Queen Square Institute of Neurology, University College London, London WC1N 3AR, UK; Department of Neurology, Memory and Aging Center, University of California, San Francisco CA 94158, USA; Department of Neurology, Memory and Aging Center, University of California, San Francisco CA 94158, USA; Cambridge University Department of Clinical Neurosciences, Cambridge University Hospitals NHS Trust, Cambridge CB2 0QQ, UK; Cambridge Brain Bank, Cambridge CB2 0QQ, UK; Centre for Medical Image Computing, Department of Computer Science, University College London, London WC1V 6LJ, UK; Centre for Medical Image Computing, Department of Computer Science, University College London, London WC1V 6LJ, UK; Centre for Medical Image Computing, Department of Computer Science, University College London, London WC1V 6LJ, UK; Cambridge University Department of Clinical Neurosciences, Cambridge University Hospitals NHS Trust, Cambridge CB2 0QQ, UK; Medical Research Council Cognition and Brain Sciences Unit, Cambridge, UK; Department of Clinical and Movement Neurosciences, University College London Queen Square Institute of Neurology, London WC1N 3BG, UK; Movement Disorders Centre, University College London Queen Square Institute of Neurology, London WC1N 3BG, UK; Department of Neurology, Memory and Aging Center, University of California, San Francisco CA 94158, USA; Dementia Research Centre, Department of Neurodegenerative Disease, University College London Queen Square Institute of Neurology, University College London, London WC1N 3AR, UK; Centre for Medical Image Computing, Department of Computer Science, University College London, London WC1V 6LJ, UK; Department of Informatics, University of Sussex, Brighton BN1 9RH, UK

**Keywords:** subtype and stage inference, disease progression, corticobasal syndrome, biomarkers, machine learning

## Abstract

Although the corticobasal syndrome was originally most closely linked with the pathology of corticobasal degeneration, the 2013 Armstrong clinical diagnostic criteria, without the addition of aetiology-specific biomarkers, have limited positive predictive value for identifying corticobasal degeneration pathology in life. Autopsy studies demonstrate considerable pathological heterogeneity in corticobasal syndrome, with corticobasal degeneration pathology accounting for only ∼50% of clinically diagnosed individuals. Individualized disease stage and progression modelling of brain changes in corticobasal syndrome may have utility in predicting this underlying pathological heterogeneity, and in turn improve the design of clinical trials for emerging disease-modifying therapies. The aim of this study was to jointly model the phenotypic and temporal heterogeneity of corticobasal syndrome, to identify unique imaging subtypes based solely on a data-driven assessment of MRI atrophy patterns and then investigate whether these subtypes provide information on the underlying pathology. We applied Subtype and Stage Inference, a machine learning algorithm that identifies groups of individuals with distinct biomarker progression patterns, to a large cohort of 135 individuals with corticobasal syndrome (52 had a pathological or biomarker defined diagnosis) and 252 controls. The model was fit using volumetric features extracted from baseline T1-weighted MRI scans and then used to subtype and stage follow-up scans. The subtypes and stages at follow-up were used to validate the longitudinal consistency of the baseline subtype and stage assignments. We then investigated whether there were differences in associated pathology and clinical phenotype between the subtypes. Subtype and Stage Inference identified at least two distinct and longitudinally stable spatiotemporal subtypes of atrophy progression in corticobasal syndrome; four-repeat-tauopathy confirmed cases were most commonly assigned to the *Subcortical* subtype (83% of individuals with progressive supranuclear palsy pathology and 75% of individuals with corticobasal-degeneration pathology), whilst those with Alzheimer’s pathology were most commonly assigned to the *Fronto-parieto-occipital subtype* (81% of individuals). Subtype assignment was stable at follow-up (98% of cases), and individuals consistently progressed to higher stages (100% stayed at the same stage or progressed), supporting the model’s ability to stage progression. By jointly modelling disease stage and subtype, we provide data-driven evidence for at least two distinct and longitudinally stable spatiotemporal subtypes of atrophy in corticobasal syndrome that are associated with different underlying pathologies. In the absence of sensitive and specific biomarkers, accurately subtyping and staging individuals with corticobasal syndrome at baseline has important implications for screening on entry into clinical trials, as well as for tracking disease progression.

## Teaser

Scotton *et al*. report distinct subtypes of atrophy progression in corticobasal syndrome identified using machine learning analysis of structural MRI scans. These subtypes correlate with underlying pathologies, such as primary tauopathies and Alzheimer's disease, with important implications for screening on entry into clinical trials, and tracking disease progression.

## Graphical Abstract

**Graphical Abstract:**
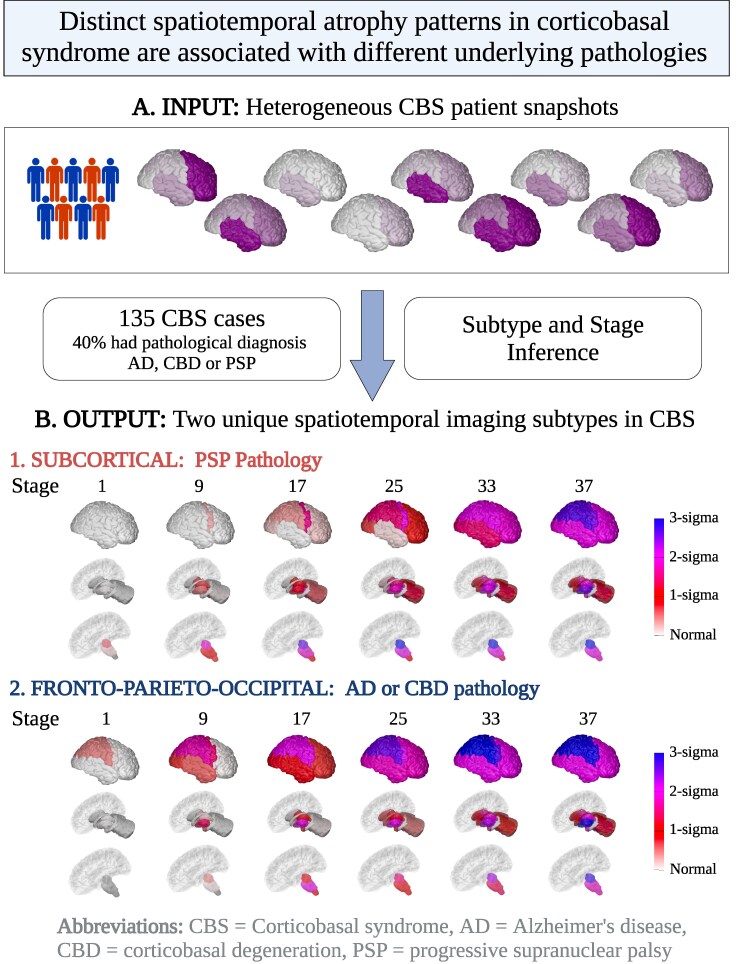

## Introduction

The corticobasal syndrome (CBS) is characterized by a progressive asymmetric akinetic-rigid syndrome and cortical features including apraxia, cortical sensory loss and cognitive dysfunction.^1^ Although CBS was first described in individuals with corticobasal degeneration (CBD) pathology at post-mortem,^2^ autopsy studies demonstrate considerable underlying pathological heterogeneity in those who present clinically with CBS.^3^ CBD pathology only accounts for ∼50% of all clinically diagnosed CBS patients,^4^ with the others usually having other primary tauopathies [such as progressive supranuclear palsy (PSP), Pick’s disease (PiD), and globular glial tauopathy (GGT)], transactive response DNA binding protein 43 (TDP-43) proteinopathy, and Alzheimer’s disease pathology^3,5-8^ This underlying pathological heterogeneity explains why the Armstrong Criteria^9^ have a limited positive predictive value for identifying underlying CBD pathology in life.^8^

An atypical posterior variant of Alzheimer’s disease was defined in the 2014 diagnostic criteria,^10^ but it is only with the emergence of amyloid and tau PET tracers, alongside CSF and now plasma biomarkers for Alzheimer’s disease,^11,12^ that CBS associated with versus without Alzheimer’s disease pathology^13^ can be identified. Biomarkers that are positive indicators for 4R tau (CBD, PSP, GGT), 3R tau (PiD) and TDP-43 are less well developed in comparison, and although various tests are currently under investigation in the research setting, none are yet validated for routine clinical use. Structural MRI studies of CBS cases with post-mortem pathology show that at the group level there are differences in the cross-sectional pattern of atrophy between some pathologies (CBD, PSP, TDP-43 and Alzheimer’s disease), and between those with CBS associated with versus without amyloid pathology. It is unclear, however, to what extent such findings are driven by differences in disease stage at time of MRI versus pathology-specific differences, given that the studies either do not correct for underlying disease stage^5,14^ or use Mini-mental State Examination (MMSE) as a proxy for stage.^15^ Grouping individuals based on cross-sectional MRI atrophy patterns without fully accounting for disease stage may be suboptimal, as different atrophy patterns may occur within the same subgroup due to individuals being at different disease stages.^16^ Predicting the pathology underlying CBS when Alzheimer’s disease biomarkers are negative, is therefore difficult due to the lack of both clinico-pathological correlation and specific biomarkers. Developing individualized disease progression models of pathological brain changes in CBS that predict this underlying heterogeneity will be critical to the success of clinical trials for emerging disease-modifying therapies^17-20^

In recent years, advances in machine-learning have provided tools to disentangle this phenotypic (clinical subtype) and temporal (pathological stage) heterogeneity. One such algorithm, Subtype and Stage Inference (SuStaIn),^21^ combines disease progression modelling with clustering to identify probabilistic data-driven disease subtypes with distinct temporal progression patterns, using only cross-sectional data. SuStaIn was originally applied to structural MRIs in Alzheimer’s disease whilst more recent work includes identifying distinct patterns of tau and amyloid accumulation in Alzheimer’s disease using PET data.^22,23^ The clinical, anatomical and pathological heterogeneity of CBS makes it ideally suited to modelling using SuStaIn.

The aim of this study was to uncover imaging subtypes of CBS based solely on a data-driven assessment of atrophy patterns, to test the hypothesis that modelling disease subtype and stage jointly would provide information on the underlying pathology. To this end, we used the SuStaIn algorithm with cross-sectional structural MRI data from a large international cohort of clinically diagnosed CBS patients. We further compared the clinical phenotypes and associated pathology in each SuStaIn subtype to gain insight into the relationship between atrophy, underlying pathology and clinical features.

## Materials and methods

### Study cohorts and clinical data

MRI and clinical data from individuals with a clinical diagnosis of ‘possible’ or ‘probable’ CBS per Armstrong’s 2013 criteria^9^ were collected from seven main cohorts: the 4R Tauopathy Imaging Initiative Cycle 1 (4RTNI 1; ClinicalTrials.gov: NCT01804452),^24,25^ the 4R Tauopathy Imaging Initiative Cycle 2 (4RTNI 2; ClinicalTrials.gov: NCT02966145), the davunetide randomized control trial (DAV; ClinicalTrials.gov: NCT01056965),^26^ the salsalate clinical trial (SAL; ClinicalTrials.gov: NCT02422485),^27^ the young plasma clinical trial (YP; ClinicalTrials.gov: NCT02460731),^27^ the PROgressive Supranuclear Palsy CorTico-Basal Syndrome Multiple System Atrophy Longitudinal Study (PROSPECT; ClinicalTrials.gov: NCT02778607),^28^ and the University College London Dementia Research Centre (UCL DRC) FTD cohort. Controls were collected from three cohorts with equivalent available data; PROSPECT, the UCL DRC FTD cohort and the Frontotemporal Lobar Degeneration Neuroimaging Initiative dataset (FTLDNI). Information pertaining to the recruitment, diagnostic criteria and MRI scanner acquisition protocols has been described previously.^29,30^ Appropriate ethical approval was acquired through application to each of the individual trial and research ethics committees.

For study inclusion, all participants needed to have, as a minimum, a baseline T1-weighted volumetric MRI on a 1.5 or 3 Tesla scanner, and basic demographic data (sex and age at time of scan). Clinical rating scale scores [PSP rating scale, Unified Parkinson Disease Rating Scale-III (UPDRS), Schwab and England Activities of Daily Living scale (SEADL) and Montreal Cognitive Assessment (MoCA) or MMSE at baseline and follow-up], pathology at autopsy, CSF Alzheimer’s disease biomarker positivity (Aβ1–42, tau and ptau), amyloid PET positivity (with florbetaben, florbetapir, or Pittsburgh Compound-B) and follow-up scans were also included if available. Amyloid PET scans were collected at participating 4RTNI-2 centres and positivity was defined by expert visual read by certified staff.

As detailed in previous work,^29^ original trial analyses failed to show any treatment effect (including no change in volumetric MRI measurements) in the SAL, YP and DAV trials, so data were combined from each study’s treatment and placebo arms. Longitudinal data were used to validate the consistency of SuStaIn’s subtype and stage assignments at follow-up.

Multiple Imputation via Chained Equations package (mice) was used to impute missing observations in individual PSP rating clinical subscores, when at least 80% of the assessment was complete.^31^ Given the PROSPECT and 4RTNI2 trials only assessed cognitive function using the MOCA (as opposed to the MMSE for the other trials), raw MOCA scores were converted to MMSE scores using the method first introduced by Lawton *et al*.^32^

### MRI acquisition and image processing

The MRI acquisition protocols and image processing pipeline have been described in detail in previous work.^29,30^ To summarize, cortical and subcortical structures were automatically parcellated using geodesic information flows algorithm (GIF),^33^ a multi-atlas segmentation propagation approach. Subregions of the cerebellum were parcellated using GIF based on the Diedrichsen atlas,^34^ and the brainstem structures were subsequently segmented using a version of the brainstem module available in FreeSurfer, customized to accept the GIF parcellation of the whole brainstem as input.^35^ Volumes for 24 grey matter regions were calculated: four brainstem [medulla, pons, superior cerebellar peduncle (SCP) and midbrain], three cerebellar (cerebellar cortex, dentate nucleus and vermis), eight subcortical [thalamus, globus pallidus (GP), caudate, putamen, ventral diencephalon (DC), hippocampus, amygdala and nucleus accumbens (NA)] and nine cortical (basal forebrain, cingulate, corpus callosum, frontal anterior, frontal posterior, insula, temporal, parietal and occipital) regions. A list of GIF subregions included in each cortical region is detailed in Supplementary Table 1. Total intracranial volume (TIV) was calculated using SPM12 v6225 (Statistical Parametric Mapping, Wellcome Trust Centre for Neuroimaging, London, UK) running in MATLAB R2012b (Math Works, Natick, MA, USA).^36^ All segmentations were visually inspected to ensure accurate segmentation. Regional volumes were corrected for scanner magnetic field strength (1.5T or 3T), scanner manufacturer (General Electric or Siemens), sex, age at baseline scan and TIV, by performing a linear regression on the control population and then propagating this model to the CBS population, to generate covariate-adjusted regional volumes.

We carried out pairwise comparisons between healthy controls and cases at baseline visit and selected covariate adjusted regional volumes (from the 24 listed in the previous section) where the difference between the two groups was associated with a moderate to large effect size (Cohen’s *d* effect size of 0.6 for standardized mean differences between the cases and controls). This resulted in the selection of 19 regions of interest (ROI) that were then included in downstream analysis (Supplementary Table 2); four brainstem (medulla, pons, SCP and midbrain), two cerebellar (cerebellar cortex and dentate nucleus), six subcortical (thalamus, GP, caudate, putamen, ventral DC, and amygdala) and seven cortical (corpus callosum, frontal anterior, frontal posterior, insula, temporal, parietal and occipital) regions. Regions that had a right and left label were combined (volumes summed). Covariate-adjusted volumes for these 19 ROIs were converted into *z* scores relative to the control group (see Supplementary Materials for more detail).

### Subtype and stage inference

SuStaIn is a probabilistic machine learning algorithm that simultaneously clusters individuals into groups (subtypes) and infers a trajectory of change associated with each group; that trajectory defines the disease stage (degree of disease progression within a subtype) of each individual within the corresponding group. Detailed formalization of SuStaIn has been published previously,^21^ and more detail on the algorithm and how it was applied to the data in this study is provided in the Supplementary Materials. A summary of the *Z*-score settings, MCMC iterations and number of random starting sequences used for the SuStaIn algorithm are provided in Supplementary Table 3.

The trained model was used to calculate the probability that each individual falls at each stage of each subtype, and individuals were assigned to their maximum likelihood subtype and stage (as per Young *et al*.^21^). Subtype progression patterns identified by SuStaIn were visualized using BrainPainter,^37^ which was modified to include brainstem segmentations.

### Statistical analysis

Individuals assigned to SuStaIn stage 0 (i.e. no atrophy on imaging compared to controls) were labelled as ‘*normal appearing*’. All other individuals were labelled as ‘*subtypable*’ and we assigned these to their most probable subtype and stage. In addition, CBS cases were stratified by likely underlying pathology into CBS-PSP, CBS-CBD and CBS-AD. While CBS-PSP and CBS-CBD were diagnosed by post-mortem pathology, cases were assigned to CBS-AD category either by post-mortem pathology, or if they had a positive Alzheimer’s disease biomarker in life (raised CSF Tau/A-Beta 1–42 ratio or positive Amyloid PET noting that amyloid positive biomarker status denotes presence of Alzheimer pathology not absence of CBD or PSP-pathology, and co-incidental amyloid positivity is expected to rise with age). All other cases without a post-mortem diagnosis or a positive Alzheimer’s disease biomarker were assigned as CBS-Indeterminate (CBS-IDT). Software and packages used to conduct analyses are described in the Supplementary Materials. All analyses were performed either in R (version 4.0.5) or Python (version 3.7.6).

### Baseline characteristics

We performed pairwise comparisons of baseline characteristics between all CBS cases and controls, CBS pathological diagnosis (CBS-CBD, CBS-PSP, CBS-AD and CBS-IDT) versus all CBS cases, and each CBS pathology grouping against each other, using two-tailed unpaired *t*-tests for continuous variables and $\chi^{2}$ tests for categorical variables. Statistical significance was reported at a level of *P*<0.05, both uncorrected for and corrected for multiple comparisons (Bonferroni correction).

### Association between subtype assignment and covariates

We tested for any residual association between covariates (scanner magnetic field strength, scanner manufacturer, sex, age at scan, total intracranial volume, SuStaIn stage) and SuStaIn subtype, by fitting a binomial logistic regression model to the two-subtype data, and a multinomial logistic regression to the three-subtype data. To confirm that age effects on regional brain volumes had been successfully regressed out, linear models were fit to assess for any residual association between individual covariate adjusted regional volumes and age at scan.

### Subtype characterization

First, we assessed the overall differences between subtypes independently of stage, excluding individuals assigned as normal appearing (stage 0). Two-tailed unpaired *t*-tests were performed for continuous variables and $\chi^{2}$ tests for categorical variables followed by *post hoc* pairwise comparisons for CBS pathology versus SuStaIn subtype.

To test for associations between clinical scores (PSP rating scale, UPDRS-III, SEADL and MMSE) and SuStaIn subtype, a linear mixed-effects model was fit to the data. Subject Id was modelled as a random effect (random intercept) due to some subjects having two MRI scans at different time points. SuStaIn subtype and stage, age, and sex were accounted for by fitting a linear mixed effects model [clinical score ∼ subtype + stage + (1 | ID) + AAS + sex] for each clinical test score. Significance was calculated using the *lmerTest* package,^38^ which applies Satterthwaite’s method to estimate degrees of freedom and generate *P*-values for mixed models. Statistical significance was reported at a level of *P* < 0.05, and at the Bonferroni corrected level of *P* < 0.005 for demographic variables (11 items) and clinical scores (10 variables), to account for multiple comparisons.

To assess average stage by clinical syndrome by SuStaIn subtype, we performed a one-way ANOVA (mean stage ∼ CBS pathology + sustain baseline subtype) with the *aov()* function from the *stats* package (version 3.6.2). Tukey *post hoc* significant differences were then calculated to identify the level of significance.

Next, we tested for differences in all 24 baseline regional volumes of interest between the different SuStaIn subtypes using two-tailed unpaired *t* tests, with statistical significance reported at a level of *P* < 0.05, both uncorrected for and corrected for multiple comparisons (Bonferroni correction). The rationale for using all regional volumes (24 rather than the 19 used in model fitting) was to investigate what the overall pattern of atrophy was for each subtype at baseline.

Finally, to assess for the degree of asymmetry, we tested for brain volume differences between left and right hemispheres at baseline using a laterality index (LI). This was defined as the absolute difference between left and right volumes divided by the total brain volume, multiplied by 100. We then then applied *t* tests on this index between the different subtypes for both the two- and three-subtype model, between the baseline and follow-up scans for each subtype in each model and finally between the different pathological groups. All *P*-values were Bonferroni corrected for multiple comparisons.

### Longitudinal validation

We used the longitudinal imaging data to validate the stability of subtypes, and to assess stage progression, based on the hypothesis that individuals should remain assigned to the same subtype but advance to higher stages over time (or at least remain at the same stage). Subtype stability was defined as the proportion of individuals that were assigned to the same subtype at follow-up(s) or progressed from stage 0 (normal appearing) to a higher stage and subtype (i.e. became subtypable). To assess stage progression, SuStaIn stage at baseline and follow-up(s) was compared for all individuals and the proportion of individuals that either advanced to a higher stage or stayed at the same stage at follow-up was calculated.

## Results

### Demographics

Table 1 summarizes the key baseline demographic and clinical features for CBS cases and controls included in this study. In total, this study included 500 MRI images from a total of 387 individuals; 135 had a clinical diagnosis of CBS, with 69 individuals having a total of 113 follow-up scans, and 252 controls. Of the 69 individuals that had follow-up, 27 (39%) had one follow-up scan, 40 (58%) had two follow-up scans, and two (3%) had three follow-up scans. For each individual, follow-up scan(s) were performed on the same MRI scanner as the original baseline scan, and the mean (SD) time interval from baseline to final follow-up scan was 1.04 years (±0.46). Of those diagnosed with CBS, 52 (39%) received a pathological or biomarker-based diagnosis: 12 were CBS-CBD, 6 were CBS-PSP, 34 were CBS-AD and 83 were CBS-IDT. There were no data available on co-pathologies in those that received a pathological diagnosis. Pathology group comparisons on the Laterality index also did not identify any significant differences in asymmetry, either between pathology groups or between baseline and follow-up within pathology groups. However, there was a trend to decreasing asymmetry in the CBS-AD group (baseline 2.5 SD 1.6 versus follow-up 1.7 1.0, *t* = 2.3, adj. *P* = 0.09) and the CBS-PSP group (baseline 1.9 SD 1.0 versus follow-up 1.6 1.4, *t* = 0.5, adj. *P* = 1.0), with a suggestion that asymmetry increases in the CBS-CBD group (baseline 1.8 SD 1.0 versus follow-up 2.3 0.8, *t* = −1.5, adj. *P* = 0.57).

**Table 1:** Baseline clinical and demographic data (by pathology)

	Controls	All CBS	CBS-CBD	CBS-PSP	CBS-AD	CBS-IDT
Baseline, *n* (fu visits)	252	135 (113)	12 (13)	6 (5)	34 (26)	83 (69)
Sex, % female	57%	51%	50%	83%	38%	54%
Age first scan, y	62.3 (9.2)^c^	66.4 (7.7)^c^	65.2 (7.0))	70.4 (5.7)	66.5 (7.7)	66.3 (8.0)
Age first symptom, y^a^		61.5 (8.7)	64.8 (6.2)	60 (2.83)	61.5 (8.0)	61.2 (9.4)
Disease duration, y^a,b^		4.9 (2.9)	3.4 (1.6)^d,e,f^	4.3 (1.6)	4.9 (3.2)^e^	5.2 (2.9)^f^
PSP rating scale score		26.3 (13.9)	26.7 (15.0)	33.8 (8.7)	24.9 (12.8)	26.5 (14.6)
History		5.6 (3.2)	6.7 (3.9)	6.8 (4.5)	5.0 (2.6)	5.7 (3.3)
Mentation		3.1 (2.8)	2 (1.5)	3.8 (1.3)	3.7 (3.4)	3.0 (2.4)
Bulbar		1.7 (2.1)	0.9 (1.2)^f^	1.0 (0.8)	1.4 (2.3)	2.0 (2.1)^f^
Ocular motor		2.3 (3.5)	2.9 (3.8)	3.8 (2.2)	1.7 (2.2)	2.4 (4.0)
Limb motor		7.7 (3.7)	7.7 (3.7)	9.2 (2.1)	7.3 (3.7)	7.7 (3.9)
Gait and midline		5.9 (5.0)	6.6 (5.1)	9.3 (7.2)	5.7 (5.2)	5.7 (4.9)
SEADL		57.8 (25.5)	55.7 (20.7)	42.5 (54.4)	53.2 (27.6)	61.0 (24.7)
UPDRS-III		32.0 (17.2)	34.3 (13.0)	47.2 (26.4)	31.2 (20.2)	31.0 (15.7)
MMSE		23.8 (5.9)	23.3 (7.5)	19.2 (8.6)	22.0 (7.4)	25.0 (4.3)

Values are mean (SD), apart from Sex % female, Baseline *n* (*n* follow-up visits), Pathology *n* (% PSP). Pairwise comparisons between groups were performed using *t* tests for continuous variables and χ^2^ tests for categorical variables.

^a^Note incomplete data for disease duration/age at first symptom.

^b^Time from first symptom to first scan.

^c^All CBS vs Controls. Statistically significant at *p* < 0.05, corrected for multiple comparisons.

^d^CBS [pathology group] vs All CBS. Statistically significant at *p* < 0.05, corrected for multiple comparisons.

^e^CBS-CBD vs CBS-AD. Statistically significant at *p* < 0.05, uncorrected for multiple comparisons.

^f^CBS-CBD vs CBS-IDT. Statistically significant at *p* < 0.05, uncorrected for multiple comparisons.

AD, Alzheimer's disease; CBD, corticobasal degeneration; IDT, indeterminate pathology; MMSE, Mini–Mental State Examination; PSP, progressive supranuclear palsy; SEADL, Schwab and England Activities of Daily Living; UPDRS, Unified Parkinson's Disease Rating Scale.

Overall, the CBS cases had an older average age at time of first scan compared with controls (66.4 years, SD 7.7 versus 62.3 years, SD 9.2, *P* < 0.05, corrected for multiple comparisons), though were matched for sex. Disease duration (defined as time from symptom onset to scan) at time of first scan was lower in the CBS-CBD group compared with CBS-AD and CBS-IDT (3.4 years, SD 1.6 versus 4.9 years, SD 3.2 versus 5.2 years, SD 2.9, *P* < 0.05 for all uncorrected for multiple comparisons) although this did not survive Bonferroni correction.

Regarding clinical scores, the only statistically significant difference between pathology groups was in the Bulbar sub-score of the PSP rating scale which was lower in the CBS-CBD group compared to CBS-IDT (0.9, SD 1.2 versus 2.0, SD 2.1, *P* < 0.05 uncorrected for multiple comparisons). There was also no difference between the SEADL and MMSE scores between pathological groups.

### Spatiotemporal subtypes of CBS

Given CBS is such a rare disease (3/100 000 estimated prevalence^39,40^), we trained SuStaIn using CBS cases only, based on the rationale that it is very unlikely any of our controls had asymptomatic CBS. Indeed, it is more likely that the controls would have a more common neurodegenerative disorder such as Alzheimer’s disease, which may confound subtype and stage inference, further supporting the exclusion.

We started with the hypothesis that there would be three distinct subtypes of atrophy in the CBS cohort each associated with a different underlying pathology. Comparing the out-of-sample log likelihoods and CVIC for the three-subtype model and the two-subtype model demonstrated that the two-subtype model (Supplementary Fig. 1A) best described the data with the lowest CVIC (Supplementary Fig. 1B). Given that the study was likely to be underpowered with only 135 cases, we decided to investigate both the two-subtype and the three-subtype models to compare the disease progression patterns and clinical phenotypes.

### Two-subtype model

**Figure 1:**
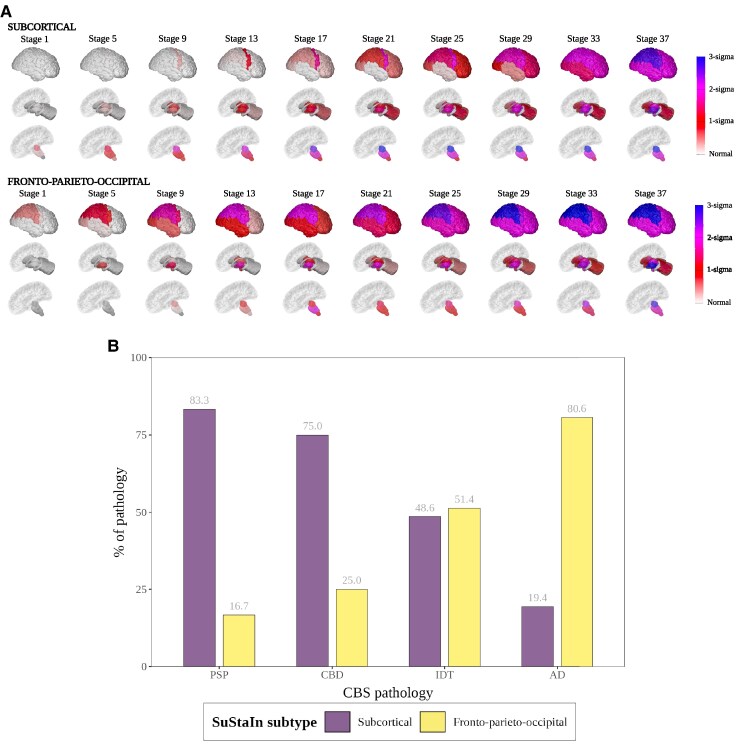
**Two-subtype model of atrophy progression in CBS identified by subtype and stage inference (SuStaIn).** (**A**) Spatial distribution and severity of atrophy at each SuStaIn stage by Subtype. Each row (Subcortical top, Fronto-parieto-occipital bottom) represents a subtype progression pattern identified by SuStaIn consisting of a set of stages at which brain volumes in CBS cases reach different z-scores relative to controls. Total *n* = 123 (subcortical *n* = 56, fronto-parieto-occipital = 67). (**B**) Assignment of CBS pathology to each SuStaIn subtype. Size of bar (*x*-axis) represents percentage of cases labelled with that PSP syndrome assigned to that SuStaIn subtype (*y*-axis). Total *n* = 123 (PSP = 6, CBD = 12, IDT = 74, AD = 31). PSP = PSP pathology at post-mortem, CBD = at post-mortem, AD = AD pathology at post-mortem or a positive AD biomarker (CSF or amyloid PET) during life. Visualizations in **A** were generated using the BrainPainter software,^37^ modified to include the brainstem segmentations.

Based on the earliest MRI abnormalities seen in the SuStaIn defined trajectories, we named the first the subcortical subtype and the second the Fronto-parieto-occipital subtype [Fig. 1A and Supplementary Fig. 2 for positional variance diagrams (PVD)]. The *Subcortical* subtype (62/135, 46% of cases) starts with atrophy in the SCP of the cerebellum and the midbrain, followed by the pons, medulla, ventral DC, dentate nucleus and thalamus. The atrophy then progresses to the posterior frontal cortex and the insula, posteriorly to the parietal and occipital cortices and anteriorly to the anterior frontal cortices, before finally affecting the temporal cortices. In contrast, in the *Fronto-parieto-occipital* subtype (73/135, 54% of cases), the earliest atrophy starts in the parietal cortex and posterior frontal cortex, followed by the insula, occipital and then temporal cortex. Atrophy in the basal ganglia (putamen and GP) also occurs earlier on in this subtype than the *Subcortical* subtype, while the brainstem, thalamus and ventral DC become atrophic later in sequence.

Overall, 12 of the 135 individuals (9%) in the two-subtype model were normal appearing at baseline, and so were excluded from subtype *post hoc* analysis. Three of these individuals had a pathological diagnosis of Alzheimer’s disease (CBS-AD) and nine were CBS-IDT. Interestingly, of the nine CBS-IDT, six had negative Alzheimer’s disease biomarkers and were therefore a pathology other than Alzheimer’s disease.

The group comparisons on the laterality index did not identify significant asymmetry in the individuals assigned to the *Fronto-parietal-occipital* as compared with *Subcortical* subtype (2.2, SD 1.5 versus 2.1, SD 1.3, *t* = 0.3 *P* = 0.75). There was also no significant change in asymmetry for either subtype for baseline scans as compared to follow-up scans; *Fronto-parietal-occipital* (baseline 2.2, SD 1.5 versus follow-up 2.2, SD 1.9, *t* = −0.2 *P* = 0.82) and *Subcortical* (baseline 2.1, SD 1.3 versus follow-up 2.1, SD 1.2, *t* = −0.2 *P* = 0.82).

A binomial logistic regression model was fitted to assess for any residual association between SuStaIn subtype, SuStaIn stage, and regressed covariates (SuStaIn subtype ∼ SuStaIn stage + TIV + age at first scan + sex + scanner manufacturer/field strength). Apart from age at first scan [younger in *Fronto-parietal-occipital* subtype (*z* = 2.0, *P* = 0.04)], there was no dependency of subtype on any of the other covariates (Supplementary Fig. 3) including SuStaIn stage, which showed a similar distribution of stages across each subtype (Supplementary Fig. 4). We confirmed that despite the difference in age between cases and controls, age effects had been effectively regressed out of the regional covariate adjusted volumes for both cases (Supplementary Table 4 and Supplementary Fig. 5) and controls (Supplementary Table 5 and Supplementary Fig. 6).

### Three-subtype model

**Figure 2:**
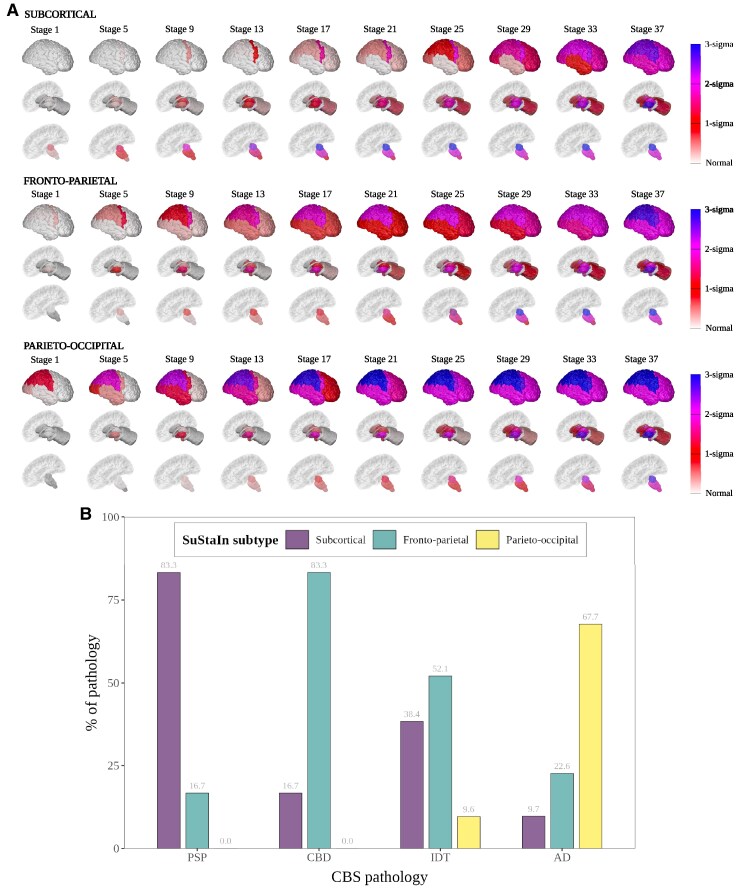
**Three-subtype model of atrophy progression in CBS identified by subtype and stage inference (SuStaIn).** (A) Spatial distribution and severity of atrophy at each SuStaIn stage by Subtype. Each row (subcortical top, fronto-parietal middle and parieto-occipital bottom) represents a subtype progression pattern identified by SuStaIn consisting of a set of stages at which brain volumes in CBS cases reach different *z*-scores relative to controls. Total *n* = 122 (subcortical *n* = 38, fronto-parietal = 56, parieto-occipital = 28). **(B)** Assignment of CBS pathology to each SuStaIn subtype. Size of bar (*x*-axis) represents percentage of cases labelled with that PSP syndrome assigned to that SuStaIn subtype (*y*-axis). Total *n* = 123 (PSP = 6, CBD = 12, IDT = 73, AD = 31). PSP = PSP pathology at post-mortem, CBD = at post-mortem, AD = AD pathology at post-mortem or a positive AD biomarker (CSF or Amyloid PET) during life.

In the three-subtype model (Fig. 2A and Supplementary Fig. 7 for the PVDs), the *Subcortical* subtype (43/135, 32% of cases) was also present with a very similar trajectory of atrophy to the *Subcortical* subtype in the two-subtype model. Of these 43 cases, 39 of them (91%) were also assigned to the *Subcortical* subtype in the two-subtype model. The second subtype we named the *Fronto-parietal* subtype (62/135, 46% of cases), which had earliest atrophy in the posterior frontal and basal ganglia regions, followed closely by the insula and parietal regions. The midbrain and thalamus were affected next followed by the temporal and occipital cortices. The third, *Parieto-occipital* (30/135, 22%) subtype, showed the most posterior atrophy with the parietal and occipital cortices affected first followed by the posterior frontal cortex and putamen, then the insula amygdala and temporal cortex.

13 of the cases (9.6% of all cases) in the three-subtype model were normal appearing (stage 0) at baseline; 12 of these were also normal appearing in the two-subtype model. Three of these had a pathological diagnosis of CBS-AD, and the other 10 were CBS-IDT. Six of the 10 CBS-IDT cases were negative for Alzheimer’s disease biomarkers. There was similar distribution of stages across each subtype (Supplementary Fig. 8).

Although there was a difference in the mean Laterality Index between the three groups at baseline [laterality index (SD) ordered from most to least asymmetry; *Parieto-occipital* 2.4 (1.7), *Subcortical* 2.2 (1.3) and *Fronto-parietal* 2.0 (1.3)], these differences did not meet statistical significance. When comparing baseline and follow-up groups only the *Parieto-occipital* subtype showed an effect with decreasing asymmetry at follow-up [baseline 2.4 (1.7) versus follow-up 1.3 (1.0), *t* = 2.9, adj.*P* = 0.02]. There was a trend to decreasing asymmetry in the *Subcortical* group [baseline 2.2 (1.3) versus follow-up 2.0 (1.6), *t* = 0.4, adj.*P* = 1.0] and increasing asymmetry in the *Fronto-parietal group* [baseline 2.0 (1.2) versus follow-up 2.6 (1.8), *t* = −2.2, adj. *P* = 0.09] though neither were statistically significant.

A multinomial logistic regression model was fitted to assess for any residual association between SuStaIn subtype, SuStaIn stage and regressed covariates (SuStaIn subtype ∼ SuStaIn stage + TIV + age at first scan + sex + scanner manufacturer/field strength). There was no dependency of subtype on any of the other covariates including SuStaIn stage (Supplementary Fig. 9).

### Longitudinal consistency of models

To validate the models’ inference of subtype longitudinal trajectories from the baseline MRI data, we tested the trained SuStaIn model’s ability to subtype and stage the follow-up MRI data. A total of 103 follow-up (103/113) scans were subtypable for both the two- and three-subtype models from a total of 63 CBS cases (47% of all CBS cases in cohort; 23 cases had one follow-up scan, 37 had two follow-up scans and two had three follow-up scans). The 10 normal appearing scans at follow-up were also normal appearing at baseline scan. The mean (SD) time interval from baseline to final follow-up scan was 1.06 years (± 0.47).

### SuStaIn subtype assignments were stable at follow-up

Overall, the two-subtype model showed the highest subtype assignment stability with 98% of those with subtypable follow-up scans (101/103) remaining in the same subtype at follow-up or progressing to a subtype from being non-subtypable at baseline (one case; Supplementary Table 6). Two cases assigned to the *Fronto-parieto-occipital* subtype switched to the *Subcortical* subtype at follow-up (both CBS-AD). The average probability with which SuStaIn assigned individuals to the subtypes at baseline was high; 0.92 (SD 0.1) for the *Subcortical* subtype and 0.94 (SD 0.1) for the *Fronto-parieto-occipital* subtype.

For the three-subtype model, 93% (96/103) of cases showed subtype assignment stability (Supplementary Table 7); five cases switched from the *Subcortical* subtype to the *Fronto-parietal* subtype (all CBS-IDT and negative for Alzheimer’s disease biomarkers) at follow-up and two switched from the *Fronto-parietal* to the *Parieto-occipital* subtype (one was CBS-AD, and the other CBS-IDT). The average probability of subtype assignment at baseline was slightly lower than the two-subtype model; 0.87 (SD 0.2) for the *Subcortical* subtype, 0.81 (SD 0.1) for the *Fronto-parietal* subtype and 0.79 (SD 0.2) for the *Parieto-occipital* subtype.

### Individuals consistently progressed to higher stages at follow-up

**Figure 3:**
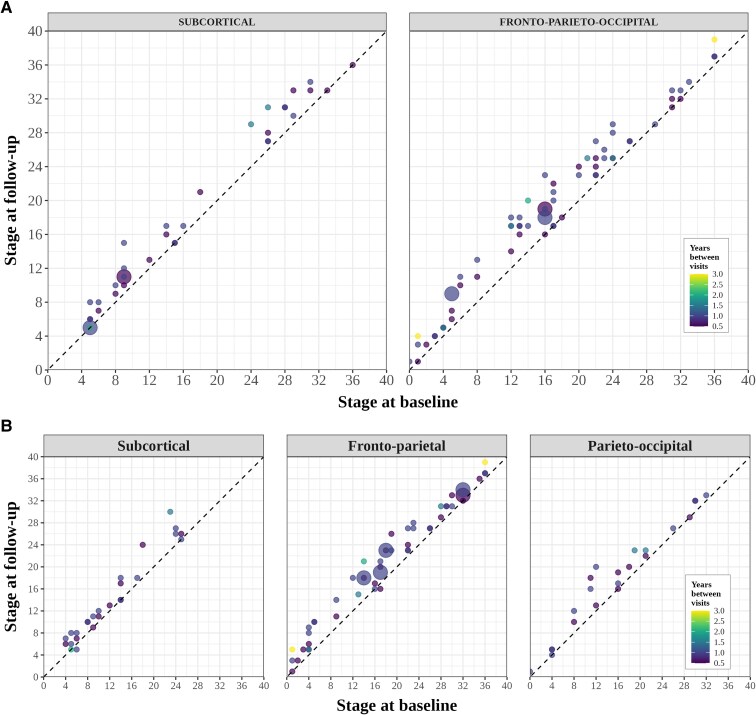
**Stage progression at follow-up visits by SuStaIn subtype.** Scatter plots of each subtype for (**A**) the two-subtype model (**B**) the three-subtype model showing predicted stage at baseline (*x*-axis) versus predicted stage at follow-up scan (*y*-axis) for those subtypable CBS cases with a follow-up scan (*n* = 103). The area of the circle is weighted by the number of scans at each point, and the colour of the circle represents the time (years) between visits.

In the two-subtype model, 100% of subtypable individuals either stayed at the same stage (15%, 15/103) or progressed to a higher stage (85%, 88/103; Fig. 3A). The *Fronto-parieto-occipital* subtype had a slightly higher percentage progressing to a higher stage at follow-up (88%, 59/67) compared with the *Subcortical* subtype (81%, 29/36).

In the three-subtype model, 98% stayed at the same stage or progressed (11%, 11/103 and 87%, 90/103, respectively; Fig. 3B). Two individuals (2%; both CBS-CBD, one assigned to the *Fronto-parietal* and one assigned to the *Subcortical* subtype) dropped one stage at follow-up.

### Subtypes were differentially enriched for underlying CBS pathologies

In the two-subtype model, the *Subcortical* subtype is associated with four-repeat Tau (4RT) pathology and the *Fronto-parieto-occipital* subtype with Alzheimer’s disease pathology (Fig. 1B). A total of 83.4% of CBS-PSP cases (5/6) and 75% of the CBS-CBD cases (9/12) were assigned to the *Subcortical* subtype, whereas 80.6% of CBS-AD cases (25/35) were assigned to the *Fronto-parieto-occipital* subtype (Table 2). There was little difference in baseline demographic and clinical scores between the two subtypes. When looking at regional unadjusted baseline volumes in the two-subtype model (Supplementary Table 8), those assigned to the *Fronto-parieto-occipital* subtype had significantly lower mean volumes in the temporal, parietal, occipital cortices compared to the *Subcortical* subtype at baseline scan. In contrast, the *Subcortical* subtype had significantly lower volumes in the midbrain, pons, SCP, dentate and the ventral DC.

**Table 2:** Comparison of demographics, pathological diagnosis, and clinical test scores between subtypes (two-subtype model)

	Subcortical	Fronto-parieto-occipital	*P-*value
All scans, *n*	62 (45.9)	73 (54.8)	
Subtypable scans, *n*	56 (45.5)	67 (54.5)	0.77^a^
Average subtype probability^b^	0.92 (0.1)	0.94 (0.1)	0.33
Sex, % female	50%	55%	0.56
Age first scan, y	68.3 (7.9)	65.4 (7.2)	0.03^c^
Age at first symptom, y^d^	64.0 (9.3)	60.3 (7.7)	0.06
Disease duration, y^d,e^	4.4 (2.7)	5.1 (2.8)	0.18
CBS pathology, *n*			-
CBS-CBD	9 (75%)	3 (25%)	
CBS-PSP	5 (83%)	1 (17%)	
CBS-AD	6 (19%)	25 (81%)	
CBS-IDT	36 (49%)	38 (51%)	<0.001^f^
PSP rating scale	27.8 (13.6)	24.8 (14.6)	0.31
SEADL	58.5 (22.7)	55.9 (28.5)	0.62
UPDRS-III	33.2 (17.7)	31.1 (17.5)	0.55
MMSE	23.8 (4.9)	23.5 (7.0)	0.82

Values are mean (SD) or *n* (%), apart from Sex = % female. Pairwise comparisons between groups were performed using *t* tests for continuous variables and χ2 tests for categorical variables.

^a^All scans vs. subtypable scans.

^b^Subtype probability = the probability of assignment for an individual case to given subtype.

^c^Statistically significant at *p* < 0.05, uncorrected for multiple comparisons.

^d^Note incomplete data for disease duration/age at first symptom.

^e^Time from first symptom to first scan.

^f^Statistically significant at *p* < 0.05, corrected for multiple comparisons.

AD, Alzheimer's disease; CBD, corticobasal degeneration; CBS, corticobasal syndrome; IDT, pathology indeterminate; MMSE, Mini–Mental State Examination; PSP, progressive supranuclear palsy; SEADL, Schwab and England Activities of Daily Living; UPDRS, Unified Parkinson's Disease Rating Scale.

In the three-subtype model, the addition of a third subtype separates CBS-CBD from CBS-PSP pathology with CBS-AD pathology predominantly assigned to a parieto-occipital subtype (Fig. 2B). In those with CBS-CBD, 83% (10/12) were assigned to the *Fronto-parietal* subtype with the remainder assigned to the *Subcortical* subtype, whilst in CBS-PSP, 83% (5/6) are assigned to the *Subcortical* subtype and 17% (1/6) to the *Fronto-parietal* subtype. Neither of the CBS-4RT pathologies (PSP and CBD) were assigned to the *Parieto-occipital* subtype. In contrast, the majority of CBS-AD cases were assigned to the *Parieto-occipital* subtype (68%, 21/31) with 22% (7/31) assigned to the *Fronto-parietal* subtype and 10% (3/31) assigned to the *Subcortical* subtype (Table 3). Comparing all regional unadjusted baseline volumes in the three-subtype model (Supplementary Table 9), the *Subcortical* subtype has the lowest volumes of the three subtypes in the midbrain, SCP, pons, dentate and the ventral diencephalon, whilst the *Parieto-occipital* subtype had the lowest volumes in the temporal, parietal, occipital cortices and the hippocampus. The *Fronto-parietal* subtype had the lowest volumes in the amygdala, posterior frontal cortex and the basal ganglia of the 3 subtypes.

**Table 3:** Comparison of demographics, pathological diagnosis and clinical test scores between subtypes (three-subtype model)

	Subcortical	Fronto-parietal	Parieto-occipital	*P-*value
All scans, *n*	43 (32%)	62 (46%)	30 (22%)	-
Subtypable scans, *n*	38 (31%)	56 (46%)	28 (23%)	0.77^a^
Average subtype probability^b^	0.87 (0.2)	0.81 (0.1)	0.79 (0.2)	0.07
Sex, % female	58%	48%	47%	0.36
Age first scan, y	68.5 (6.6)	66.3 (8.4)	64.9 (7.2)	0.15
Age at first symptom, y^c^	63.3 (7.2)	62.8 (10.1)	58.6 (7.2)	0.15
Disease duration, y^c,d^	5.0 (3.1)	4.5 (2.7)	4.9 (2.5)	0.71
CBS pathology, *n*				*-*
CBS-CBD	2 (17%)	10 (83%)	0 (0%)	
CBS-PSP	5 (83%)	1 (17%)	0 (0%)	
CBS-AD	3 (10%)	7 (22%)	21 (68%)	
CBS-IDT	28 (38%)	38 (52%)	7 (10%)	<0.05^e^
PSP rating scale	28.5 (13.7)	26.0 15.3)	24.3 (12.6)	0.55
SEADL	57.8 (22.2)	59.8 (25.7)	51.2 (31.1)	0.43
UPDRS	35.0 (18.5)	30.6 (15.8)	31.5 (20.1)	0.58
MMSE	23.9 (4.6)	25.3 (4.5)	20.1 (8.8)	<0.05^e^

Values are mean (SD) or n (%), apart from sex = % female. Group comparisons were performed using a linear model for continuous variables (continuous variable ∼ SuStaIn subtype) and χ2 tests for categorical variables.

^a^All scans versus subtypable scans.

^b^Subtype probability = the probability of assignment for an individual case to a given subtype.

^c^Note incomplete data for disease duration/age at first symptom.

^d^Time from first symptom to first scan.

^e^Statistically significant at *P* < 0.05, corrected for multiple comparisons.

AD, Alzheimer's disease; CBD, corticobasal degeneration; CBS, corticobasal syndrome; IDT, pathology indeterminate; MMSE, Mini-Mental State Examination; PSP, progressive supranuclear palsy; SEADL, Schwab and England Activities of Daily Living; UPDRS, Unified Parkinson's Disease Rating Scale.

### Association between stage, subtype, and clinical disease severity

We went on to assess the association between stage, subtype and clinical disease severity in the both the two- and three- subtype model, controlling for age and sex.

In the two-subtype model (Table 4), only the Gait and midline PSP rating scale subscore was different between the *Subcortical* and *Fronto-parieto-occipital* subtype (worse in the *Subcortical* subtype: *t* = −2.04, *P* = 0.04, uncorrected) those this did not survive Bonferroni correction. Worsening total PSP rating scale score (and History, Bulbar and Oculomotor subscores) and MMSE score were associated with increasing SuStaIn stage, suggesting these scores decline with disease progression in both subtypes.

**Table 4:** Comparison of adjusted clinical scores between subtypes in the two-subtype model

	SuStaIn subtype		SuStain stage			
	*t* value	*P-*value	*t* value	*P-*value	Subtype with worse score	Change with Sustain stage
PSP rating scale score						
Total	−0.63	0.27	2.32	0.02^a^		Worsens
History	−1.11	0.78	1.99	0.04^a^		Worsens
Mentation	0.61	0.55	1.38	0.17		
Bulbar	−0.35	0.72	4.00	1 × 10^−4b^		Worsens
Ocular motor	−0.62	0.54	2.46	0.02^a^		Worsens
Limb motor	0.13	0.89	−0.25	0.80		
Gait and midline	−2.04	0.04^a^	0.34	0.73	Subcortical subtype	
SEADL	−0.31	0.75	−0.94	0.34		
UPDRS-III	−0.01	0.99	0.88	0.38		
MMSE	−0.19	0.85	−4.20	5 × 10^−5b^		Worsens

Linear mixed model of clinical score ∼ subtype + stage + (1 | ID) + AAS + sex. Significance was calculated using Satterthwaite’s method to estimate degrees of freedom and generate *P*-values. Includes 226 scans (123 baseline and 103 follow-up scans and varying timepoints).

^a^Statistically significant at *P* < 0.05, uncorrected for multiple comparisons (10 items, *P* < 0.005).

^b^Statistically significant at *P* < 0.05, corrected for multiple comparisons (10 items, *P* < 0.005).

FPO, fronto-parieto-occipital; MMSE, Mini–Mental State Examination; SEADL, Schwab and England Activities of Daily Living; UPDRS, Unified Parkinson’s Disease Rating Scale.

In the three-subtype model (Table 5), the main difference to the two-subtype model was that there was no longer a significant difference in Gait and midline subscores between the subtypes, while significant differences between performance on the MMSE became apparent in the *Parieto-occipital* subtype (*t* = −3.11, *P* = 2.4 × 10^−3^).

**Table 5:** Comparison of adjusted clinical scores between subtypes in the three-subtype model

	SuStaIn subtype (fronto-parietal^c^)		SuStaIn subtype (parieto-occipital^c^)		SuStaIn stage			
	*t* value	*P-*value	*t* value	*P-*value	*t* value	*P-*value	Subtype with worse score	Change with Sustain stage
PSP rating scale score								
Total	−1.24	0.22	−0.83	0.41	2.46	0.01^a^		Worsens
History	−0.97	0.34	−0.91	0.37	2.02	0.05^b^		Worsens
Mentation	−0.57	0.57	1.42	0.16	1.44	0.15		
Bulbar	−0.05	0.96	−1.14	0.25	3.68	3.7 × 10^−4a^		Worsens
Ocular motor	−1.39	0.17	−1.58	0.12	2.72	7.6 × 10^−3a^		Worsens
Limb motor	−0.03	0.98	−0.51	0.61	0.22	0.83		
Gait and midline	−1.58	0.11	−1.07	0.29	1.72	0.09		
SEADL	0.44	0.66	−1.16	0.25	−1.13	0.26		
UPDRS-III	−0.75	0.46	−0.24	0.81	1.05	0.30		
MMSE	1.35	0.18	−3.11	2.4 × 10^−3a^	−3.79	2.4 × 10^−4a^	Parieto-occipital	Worsens

Linear mixed model of clinical score ∼ subtype + stage + (1 | ID) + AAS + sex. Significance was calculated using Satterthwaite’s method to estimate degrees of freedom and generate *P*-values. Includes 226 scans (123 baseline and 103 follow-up scans at varying timepoints).

^a^Statistically significant at *P* < 0.05, corrected for multiple comparisons (10 items, *P* < 0.005).

^b^Statistically significant at *P* < 0.05, uncorrected for multiple comparisons (10 items, *P* < 0.005).

^c^Named SuStaIn subtype compared to *Subcortical* subtype.

MMSE, Mini–Mental State Examination; SEADL, Schwab and England Activities of Daily Living; UPDRS, Unified Parkinson’s Disease Rating Scale.

## Discussion

We applied an unsupervised machine learning algorithm (SuStaIn) to a large cohort of clinically diagnosed CBS cases, uncovering imaging subtypes based solely on a data-driven assessment of cross-sectional atrophy patterns. Prior studies have retrospectively assessed both structural^5,14,15^ and FDG-PET imaging^41^ at a group level, as correlates of CBS pathology. Three of these studies^5,14,41^ took no account of disease stage in their analysis and so are limited by the inherent assumption that all subjects are at a common disease stage (no temporal heterogeneity). The study by Whitwell *et al*.^15^ uses the MMSE score as a proxy for disease stage, although MMSE may not be similarly affected across the different pathologies or for a given stage of disease. In addition, none of these clinico-pathological studies include longitudinal imaging follow-up and provide little information on the earliest regions in the brain affected by disease within the different pathological subtypes. By using SuStaIn to jointly model both disease stage and subtype simultaneously, we were able to better account for this temporal heterogeneity, highlighting the regions that are affected earliest in the disease course for each imaging subtype, whilst also providing a fine-grained staging model within each subtype that allowed staging of individual patients.

It is important to note that the model was agnostic to underlying pathology, and we only used the pathology information *post hoc*, to test the hypothesis that these imaging subtypes would provide information on the underlying pathology. In support of this hypothesis, the subtypes were differentially associated with underlying pathology; the data best supported a two-subtype model, with 4RT (PSP or CBD) confirmed cases being most commonly assigned to the *Subcortical* subtype (83% of PSP and 75% of CBD, respectively), and Alzheimer’s disease cases being most commonly assigned to the *Fronto-parieto-occipital* subtype (81% of CBS-AD cases). The *Subcortical* subtype (46% of cases) was characterized by early atrophy of the SCP, midbrain and dentate nucleus, followed by the basal ganglia, remaining brainstem structures and the thalamus, with the posterior frontal cortex being the first cortical structure to become abnormal. This early involvement of the brainstem and subcortical structures in CBS-4RT is in keeping with previous work that shows that more severe atrophy is found in these regions in CBS-PSP and CBS-CBD compared with controls and CBS-AD.^42^ In contrast, the *Fronto-parieto-occipital* subtype demonstrates earliest atrophy in the parietal region closely followed by the posterior frontal, insular and occipital cortices. The basal ganglia, similar to the *Subcortical* subtype, are involved early in the sequence, as one might expect given these individuals have presented with a cortico-basal syndrome. The fact that Alzheimer’s disease pathology is strongly assigned to this subtype is also in keeping with published clinico-pathological imaging studies, where CBS-AD demonstrates the most severe atrophy in the parietal and posterior frontal regions.^5,14,15^

The two-subtype model best explained the data in this cohort, as evidenced by the cross-validation log likelihoods and CVIC in Supplementary Fig. 1. The three-subtype model was underpowered with several of the different subtype stages only having a single individual assigned. Given our initial hypothesis that there would be at least three CBS imaging subtypes, and the fact that there was only a small decrease in model log likelihood with a third subtype added, we decided to carry out *post hoc* analyses on both the two- and the three-subtype models. Further analysis of the three-subtype model showed that adding a third subtype allowed differentiation of PSP from CBD pathology, albeit at a loss of specificity for Alzheimer’s disease pathology. Given the availability of sensitive and specific Alzheimer’s disease biomarkers, this may allow for identification of these cases that do not map to the most ‘AD-like’ subtype, thus enriching the other subtypes for 4RT pathology. PSP pathology was still strongly assigned to the *Subcortical* subtype (83.3% of cases), though 75% of CBD cases were now assigned to the new *Fronto-parietal* subtype. Neither CBS or PSP pathology were assigned to the *Parieto-occipital* subtype, which had a very similar sequence of atrophy to the *Fronto-parieto-occipital* subtype from the two-subtype model. A total of 68% of Alzheimer’s disease pathology was assigned to this *Parieto-occipital* subtype, with 23% assigned to the new *Fronto-parietal* subtype. The sequence of atrophy on the *Fronto-parietal* subtype demonstrates earliest involvement of the posterior frontal cortex and the basal ganglia with early involvement of the parietal and insula, which is consistent with imaging in autopsy confirmed CBD cases.^5,14,15^ Interestingly this subtype also showed later involvement of the temporal cortex compared to the *Parieto-occipital* subtype, another feature that has been shown to differentiate CBS-CBD from CBS-AD.^15^ In keeping with the *Parieto-occipital* subtype being more strongly associated with Alzheimer’s disease pathology, analysis of regional volumes at baseline demonstrated that the hippocampal and temporal (as well as parietal and occipital) regions were more atrophic compared to the *Fronto-parietal* subtype at presentation. Further support for this is that the MMSE was significantly lower in the *Parieto-occipital* subtype (20.1, SD 8.8, *t* = −2.3, Bonferroni corrected *P* = 0.02) compared to the other subtypes (23.9 SD 4.6, 25.3 SD 4.9 for the *Subcortical* and *Fronto-parietal* subtypes, respectively).

When comparing clinical scores between subtypes, there was minimal difference; in the two-subtype model only the Gait and midline PSP rating scale sub-score was different (lower in the *Subcortical* subtype), whilst as mentioned above only the MMSE showed a difference between subtypes in the three-subtype model (lowest in the *Parieto-occipital* subtype). This is consistent with the lack of clinical difference between the different pathology groups at baseline in our data, and in previous studies comparing CBD with CBS-AD^8,14^ and other CBS related pathologies.^15^ The lack of difference in UPDRS-III scores between different subtypes is at first glance perhaps counter-intuitive given that in the *Subcortical* subtype the basal ganglia and brainstem are affected earlier than in the other subtypes. However, this finding is at least consistent with the few studies that have compared UPDRS-III between different CBS pathology groups (Whitwell 2010, Joseph 2018, Street 2023), where no statistical difference was found. The association of these pathologies with different imaging subtypes that have different spatiotemporal patterns of atrophy (as identified by the model) could, at least in theory give rise to phenotypic differences. The lack of distinctive clinical features according to underlying pathologies in CBS could previously have been attributed to similar spatial patterns of underlying pathology (whatever that pathology may be). However, as shown by Figs 1 and 2, the pathologies are associated with different SuStain subtypes and these subtypes represent different distributions of spatiotemporal disease burden. The lack of clinical differences might therefore reflect insufficient power, asymmetry of disease, or insensitivity of the current clinical ratings scales to the discriminating features.

Overall, the trained SuStaIn models showed strong subtyping and staging capabilities. In the two-subtype model, assignments were longitudinally consistent at 101 out of 103 (98%) of follow-up visits. The two individuals who changed from the *Fronto-parietal-occipital* subtype to *Subcortical* at follow-up were only weakly assigned at baseline (0.43 and 0.58). From a staging perspective, individuals consistently moved to higher stages over time in both subtypes, with no cases dropping to a lower stage at follow-up scan. As expected, in the three-subtype model, the subtypes were slightly less stable, which likely reflects the increased uncertainty in assignment due to lower sample sizes in each cohort.

Although this study provides preliminary evidence for multiple underlying imaging subtypes of CBS, there are important limitations that must be taken into account when interpreting our results. Although we built a large imaging cohort from the perspective of CBS (135 cases with 113 follow-up visits), this is still small for a SuStaIn analysis. We decided to combine regions from the right and left hemispheres to try and reduce the number of features included in the model and so maximize power to detect subtypes with the available sample size. It is known that CBS-CBD, in particular, is characterized by asymmetric atrophy, at least later in the disease course,^15,43^ although this is not universal, and a lack of asymmetry does not exclude a diagnosis of underlying CBD pathology.^44^ By combining the right and left cortical regions we are likely to have reduced the sensitivity for detecting a ‘CBD’ like subtype in particular, as the effect size for a given region affected by CBD pathology would be diluted by the less severe atrophy in the contralateral hemisphere.

Although classifying CBS cases with positive ante-mortem Alzheimer’s disease biomarkers as CBS-AD is an established approach,^28,45^ the gold standard is to confirm Alzheimer’s disease pathology (and the absence of co-pathologies) at post-mortem. Due to the small number of pathology confirmed CBS-AD cases in our cohort (*n* = 4), we decided to enrich for CBS-AD by using available Alzheimer’s disease biomarkers from the PROSPECT and 4RTNI cohorts. However, by using Alzheimer’s disease biomarkers to classify these cases, we cannot be sure that there are not additional co-pathologies present. Indeed, a study from the Mayo Clinic brainbank reported that 6% of their CBD cases and 10% of PSP cases also met the criteria of a neuropathological diagnosis of Alzheimer’s disease,^44^ with between 86 and 89% having at least minimal Alzheimer’s disease neuropathological change. The presence of co-pathologies in some of our CBS-AD cases is a possible explanation for why over 30% of these cases were assigned to other subtypes than the *Fronto-parieto-occipital* subtype in the 3-subtype model. A related, but separate issue is the lack of pathology or amyloid biomarker data for 74 of the cases (categorized as CBS-IDT). Although the focus of this study was to identify CBS imaging subtypes and stages a priori, we wanted to test *post hoc* the assignment of the different pathologies to the these identified subtypes to test the hypothesis that joint modelling of disease stage and subtype would provide additional information on underlying pathology. The difficulty of interpreting these results is compounded by the fact that we had no data on TDP-43 pathology, which is known to account for ∼15% of cases of CBS.^44^ It is an interesting observation that of the 12 cases that were normal appearing at baseline, nine were CBS-IDT. One might speculate that given all of the cases with known 4RT pathology were subtypable that these un-subtypable cases could have a different underlying pathology such as TDP-43, or indeed multiple co-pathologies. A good test of the pathology association with subtype will be testing whether those that come to post-mortem in the future match the expected pathology based on their subtype assignment.

In conclusion, in this study we provide preliminary data-driven evidence for the existence of at least two distinct and longitudinally stable spatiotemporal subtypes of atrophy in clinically diagnosed CBS, by jointly modelling disease stage and subtype using cross-sectional structural MRI. Underlying CBS pathology is differentially associated with these subtypes giving insights into the relationship between pathology and the topographical distribution of atrophy. In addition, our model provides an intrinsic staging and subtyping mechanism by which individual patients can be more accurately stratified according to disease stage within each subtype. In the absence of sensitive and specific biomarkers for the range of different pathologies in CBS, being able to accurately subtype and stage CBS patients at baseline has important implications for screening patients on entry into clinical trials, as well as for tracking disease progression.

## Supplementary Material

fcaf066_Supplementary_Data

## Data Availability

Source data are not publicly available, but non-commercial academic researcher requests may be made to the chief investigators of the seven source studies, subject to data access agreements and conditions that preserve participant anonymity. The underlying SuStaIn model code is publicly available at https://github.com/ucl-pond/pySuStaIn.^46^

## Appendix

### 4RTNI consortium

Bradley F. Boeve

Brad C. Dickerson

Carmela M. Tartaglia

Irene Litvan

Murray Grossman*

Alexander Pantelyat

Edward D. Huey

David J. Irwin

Anne Fagan

Suzanne L. Baker

Arthur W. Toga

*Deceased

### PROSPECT consortium

Edwin Jabbari

Marte Theilmann Jensen

Danielle Lux

Riona Fumi

David P. Vaughan

Henry Houlden

Michele T. M. Hu

Peter N. Leigh
