# Supplementary material for: Distinct spatiotemporal atrophy patterns in corticobasal syndrome are associated with different underlying pathologies
Source: Brain Commun. 2025 Feb 11;7(2):fcaf066. doi: 10.1093/braincomms/fcaf066 (PMC11894806; doi:10.1093/braincomms/fcaf066)
Supplement: fcaf066_Supplementary_Data [file fcaf066_supplementary_data.docx]

### **Methods**

### **Z-scoring of data**

Covariate adjusted regional volumes for these 19 ROIs were converted into z scores relative to the control group by subtracting the mean of the control group from each patient’s ROI volume and dividing by the standard deviation of the control group. Given regional brain volumes decrease with disease progression, the z scores become negative as the disease progresses; we therefore multiplied the z scores by -1, to give positive z scores that increase with disease progression. This z scored data was then used as input to SuStaIn.

### **Subtype and Stage Inference**

In summary, each subtypes’ progression pattern is described using a piecewise linear z score model, expressing a trajectory with a series of stages, that each correspond to a single biomarker (regional brain volume in this case) reaching a new z score. The number of SuStaIn stages is determined by the number of biomarkers (the product of the number of ROIs and number of z score thresholds per ROI) provided as input. SuStaIn optimises both the subtype membership and the ordering in which different biomarkers reach different z-scores in each subtype (for example one, two or three standard deviations away from the control mean for that ROI) using a data likelihood function.

We fitted the SuStaIn model on the baseline imaging data for CBS cases; model uncertainty was estimated using 100,000 Markov Chain Monte Carlo (MCMC) iterations and in the single-cluster expectation maximisation procedure the single-cluster sequence was optimised from 24 different random starting sequences to find the maximum likelihood solution. The optimal number of subtypes was determined using information criteria calculated through ten-fold cross-validation (cross-validation information criteria; CVIC), to balance internal model accuracy with model complexity. In cases where the evidence for a more complex model (more subtypes) was not strong (defined as per Young et al.^1^ as a difference of less than 6 between CVIC and the minimum CVIC across models, or equivalently a difference of less than 3 between the out-of-sample log-likelihood and the minimum out-of-sample log-likelihood across models), we selected the less complex model (fewer subtypes) to avoid overfitting^2^.

### **Software - Packages and Functions**

Binomial logistic regression models were fit to the data using the g*lm*() function from the stats package (version 3.6.2), while t tests were performed using the *t.test*() function the R stats package (version 3.6.2). Multinomial logistic regression models were fit to data using the *multinom*() function from the R nnnet package (7.3-19). Chi square tests were performed using the *CrossTable*() function from gmodels package (version 2.18.1.1). Linear mixed effect models were fit using the data using the lme4 package^3^ (version 1.1-34). Post hoc pairwise comparisons for CBS pathology vs SuStaIn subtype were carried out using the *chisq.multicomp*() function from the RVAideMemoire R package version 0.9.83.7).

### **Consortia member affiliations**

**4RTNI Consortium**

Bradley F. Boeve - Department of Neurology, Mayo Clinic, Rochester, MN 55905, USA

Brad C. Dickerson - Departments of Neurology and Psychiatry, Frontotemporal Disorders Unit and Alzheimer's Disease Research Center, Boston Massachusetts USA.

Carmela M. Tartaglia - Tanz Centre for Research in Neurodegenerative Diseases University of Toronto, Toronto Canada.

Irene Litvan - Department of Neurosciences, University of California San Diego, La Jolla, California, USA.

Murray Grossman* - Department of Neurology, University of Pennsylvania, Philadelphia, USA.

Alexander Pantelyat - Department of Neurology, Johns Hopkins University School of Medicine, Baltimore, MD, USA.

Edward D. Huey - Department of Psychiatry and Neurology, Columbia University, New York, New York, USA.

David J. Irwin - Penn Center for Neurodegenerative Disease Research, University of Pennsylvania School of Medicine, Philadelphia, PA, USA.

Anne Fagan - Department of Neurology, Washington University School of Medicine, St Louis, MO, USA.

Suzanne L. Baker - Molecular Biophysics and Integrated Bioimaging, Lawrence Berkeley National Laboratory, Berkeley, CA, USA.

Arthur W. Toga - Laboratory of Neuro Imaging, Stevens Neuroimaging and Informatics Institute, Keck School of Medicine of USC, University of Southern California, Los Angeles, CA, United States.

*Deceased

**PROSPECT Consortium**

Edwin Jabbari, MRCP PhD – ¹Department of Clinical and Movement Neurosciences, UCL Queen Square Institute of Neurology, London, UK; ²Movement Disorders Centre, UCL Queen Square Institute of Neurology, London, UK.

Marte Theilmann Jensen, MSc - ¹Department of Clinical and Movement Neurosciences, UCL Queen Square Institute of Neurology, London, UK; ²Movement Disorders Centre, UCL Queen Square Institute of Neurology, London, UK.

Danielle Lux, MBChB FRACP - ¹Department of Clinical and Movement Neurosciences, UCL Queen Square Institute of Neurology, London, UK; ²Movement Disorders Centre, UCL Queen Square Institute of Neurology, London, UK.

Riona Fumi, MSc - ¹Department of Clinical and Movement Neurosciences, UCL Queen Square Institute of Neurology, London, UK; ²Movement Disorders Centre, UCL Queen Square Institute of Neurology, London, UK.

David P Vaughan, MRCPI - ¹Department of Clinical and Movement Neurosciences, UCL Queen Square Institute of Neurology, London, UK; ²Movement Disorders Centre, UCL Queen Square Institute of Neurology, London, UK.

Henry Houlden, FRCP, PhD - ¹Department of Clinical and Movement Neurosciences, UCL Queen Square Institute of Neurology, London, UK; ²Movement Disorders Centre, UCL Queen Square Institute of Neurology, London, UK; 3Department of Neuromuscular Diseases, UCL Queen Square Institute of Neurology, London, UK.

Michele T. M. Hu, FRCP, PhD - Division of Neurology, Nuffield Department of Clinical Neurosciences, University of Oxford, Oxford, UK.

P Nigel Leigh, FRCP, PhD - Department of Neuroscience, Brighton and Sussex Medical School, Brighton, UK.

### **Supplementary Figures**


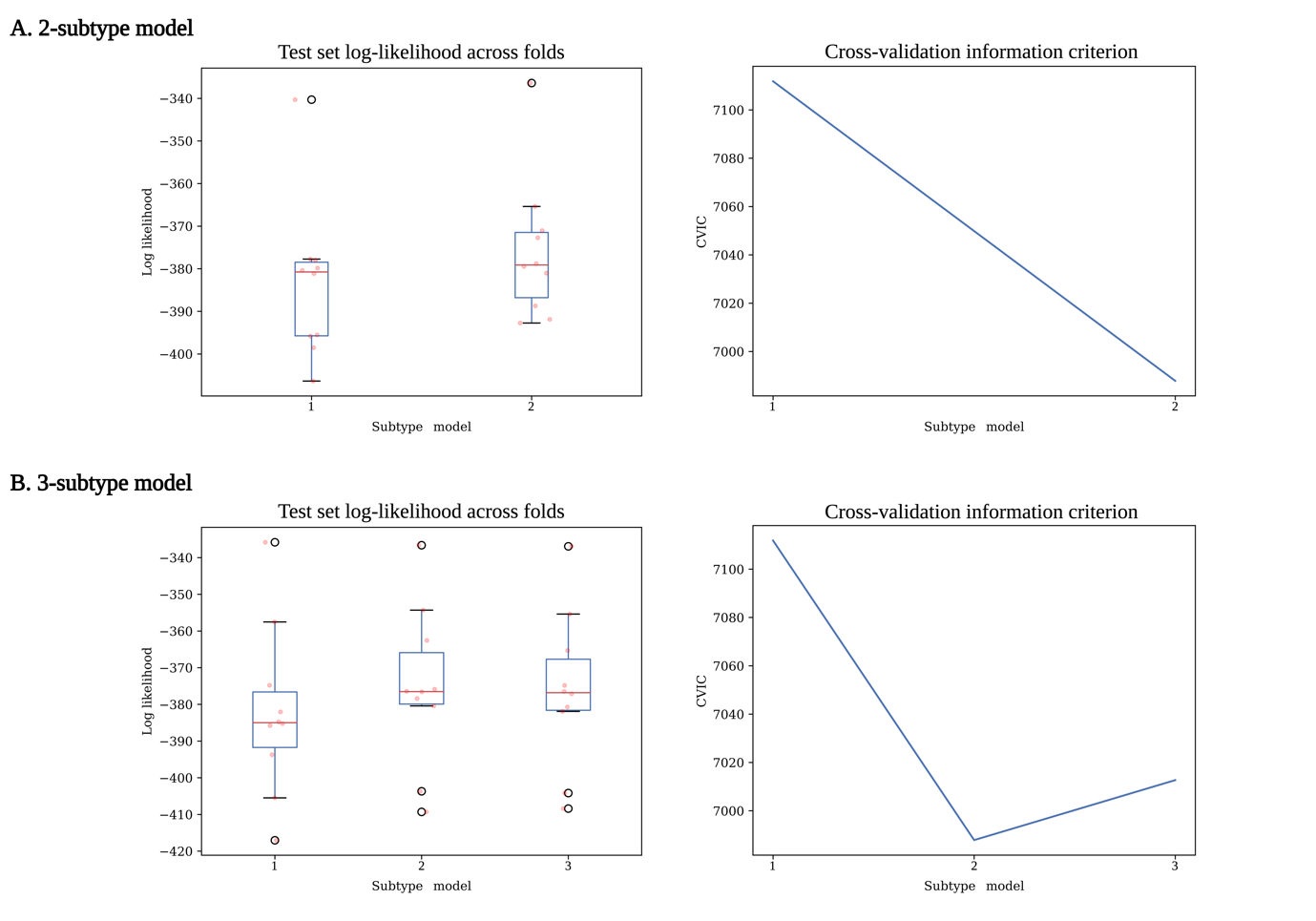


**Supplementary Figure 1 - Selecting optimal SuStaIn subtype model given data.** The plots on the left of the figure show the test set log-likelihood across ten cross validation folds for (**A**) the two-subtype model and (**B**) the three-subtype model. The plots on the right show the cross-validation information criterion (CVIC) for each of the models as detailed above. The fact that the test set log-likelihoods drop and the CVIC increased with the addition of a third subtype (**B**) suggests that the two-subtype model is the most parsimonious and best for the data. The methods used for optimal model selection are explained in more detail in the Subtype and Stage Inference section above.

**
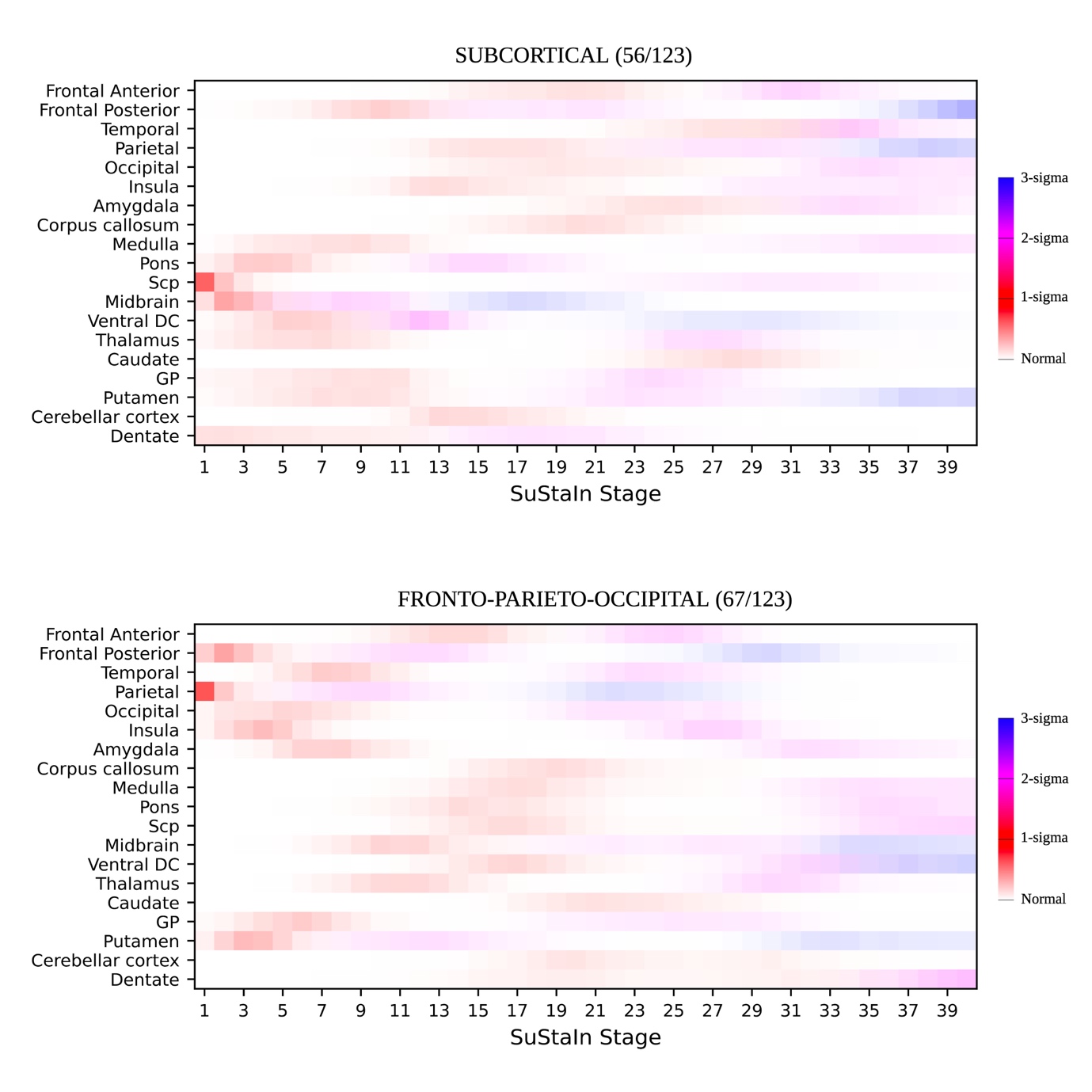
**

**Supplementary Figure 2 – Positional variance diagrams for SuStaIn subtypes in the 2-subtype model.** These represent the uncertainty in the subtype progression patterns for each region. Each region (y-axis) is shaded based on the probability a particular z score is reached at a particular SuStaIn stage (x-axis). Z scores range from zero (white), one (red), two (pink) to three (blue) as shown in the bar on the right hand side of figure. Total subtypable baseline scans =123 (*Subcortical* 56, *Fronto-parieto-occipital* 67).

**
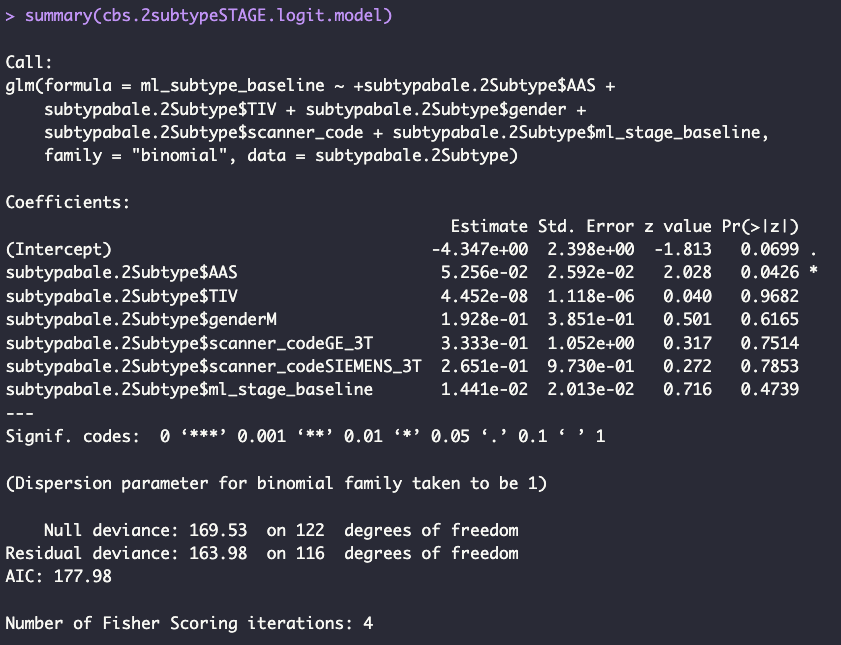
**

**Supplementary Figure 3 – Two-subtype binomial logistic regression model output.** To test for any residual association between covariates (scanner magnetic field strength, scanner manufacturer, sex, AAS, TIV, SuStaIn stage) and SuStaIn subtype, by fitting a binomial logistic regression model to the two-subtype data. Analysis was performed using the *glm*() function from the stats package (version 3.6.2) in R version 4.0.5. Total number of scans included in analysis (n) = 135. Abbreviations: TIV = total intracranial volume, AAS = age at scan.


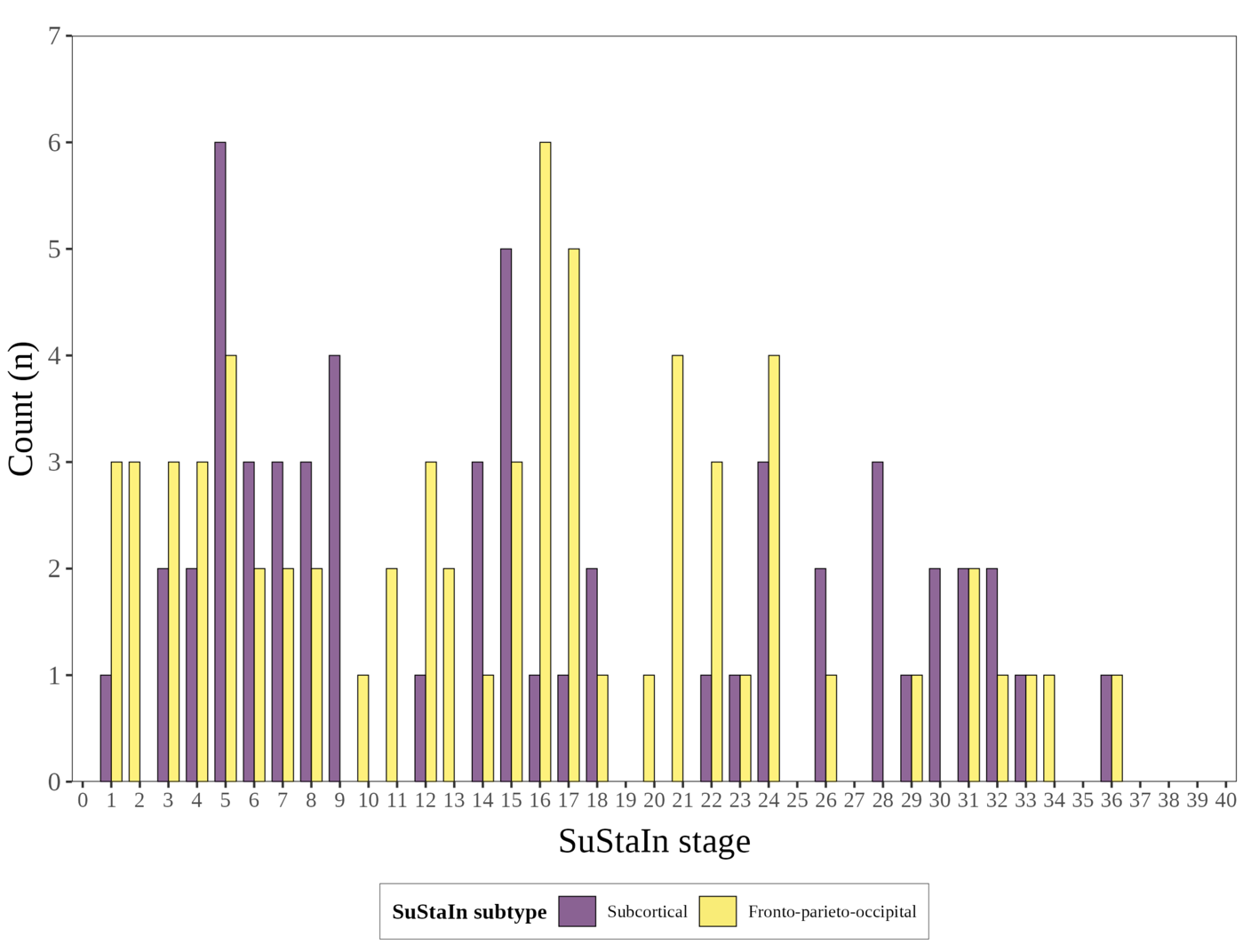


**Supplementary Figure 4 – Stage distribution by Subtype for the 2-subtype model.** Individual bars represent number (n) of cases estimated to be a particular stage by the SuStaIn algorithm, with the colour of the bar representing the subtype those cases were assigned to; purple = *Subcortical*, and yellow = *Fronto-parieto-occipital*.

**
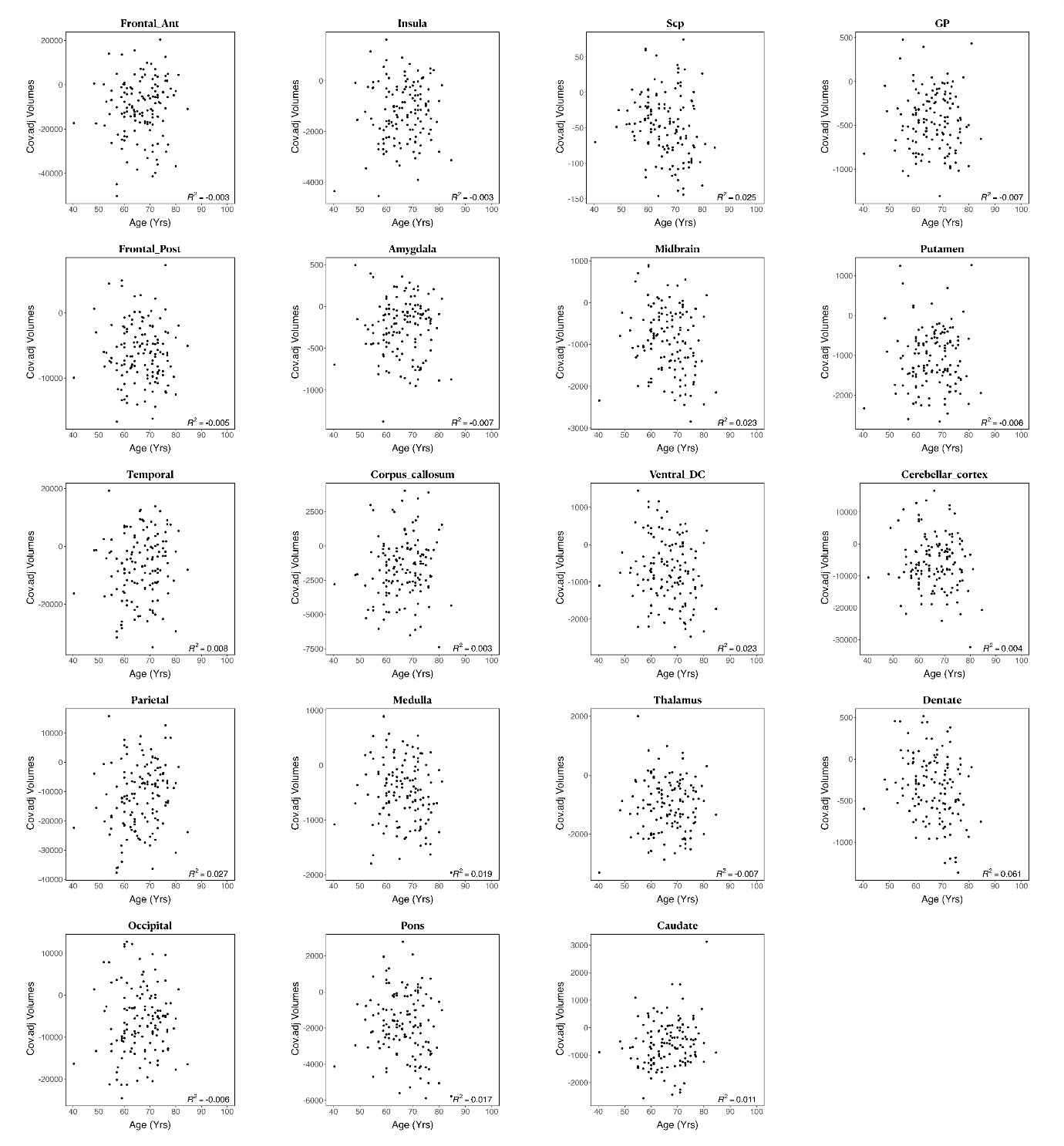
**

**Supplementary Fig. 5: Association of age at baseline scan with covariate adjusted regional volumes in cases.** Each scatter plot (19 regions in total) shows every CBS case (n=135) plotted as a function of age at baseline scan (x axis) and covariate adjusted regional volume (y axis). The R^2^ represents the proportion of the variation in the covariate adjusted regional volume that is explained by age at baseline scan (linear model = cov. adj. regional vol. ~ age at baseline scan).

**
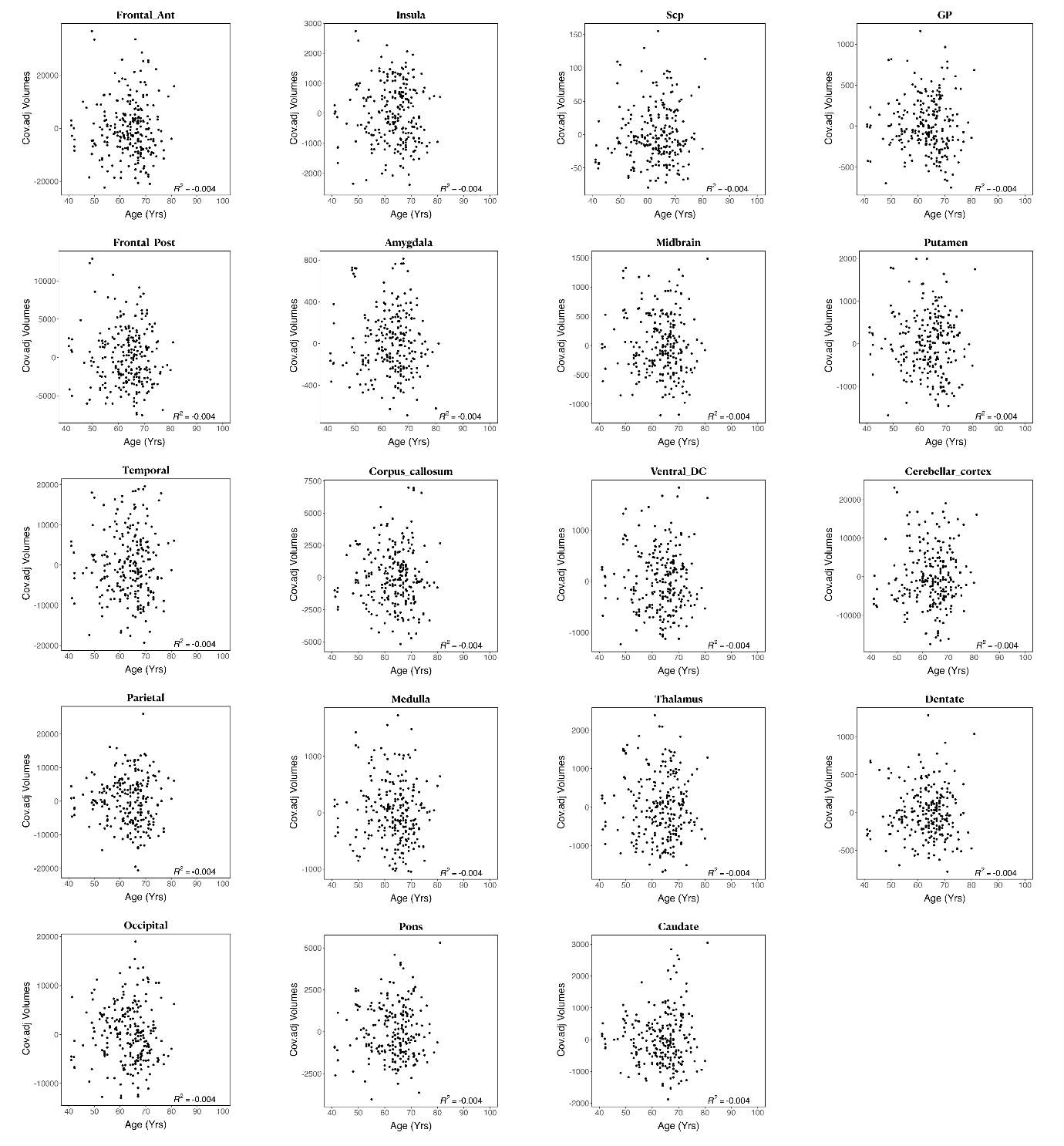
**

**Supplementary Fig. 6: Association of age at baseline scan with covariate adjusted regional volumes in controls.** Each scatter plot (19 regions in total) shows every control (n=252) plotted as a function of age at baseline scan (x axis) and covariate adjusted regional volume (y axis). The R^2^ represents the proportion of the variation in the covariate adjusted regional volume that is explained by age at baseline scan (linear model = cov. adj. regional vol. ~ age at baseline scan)

**
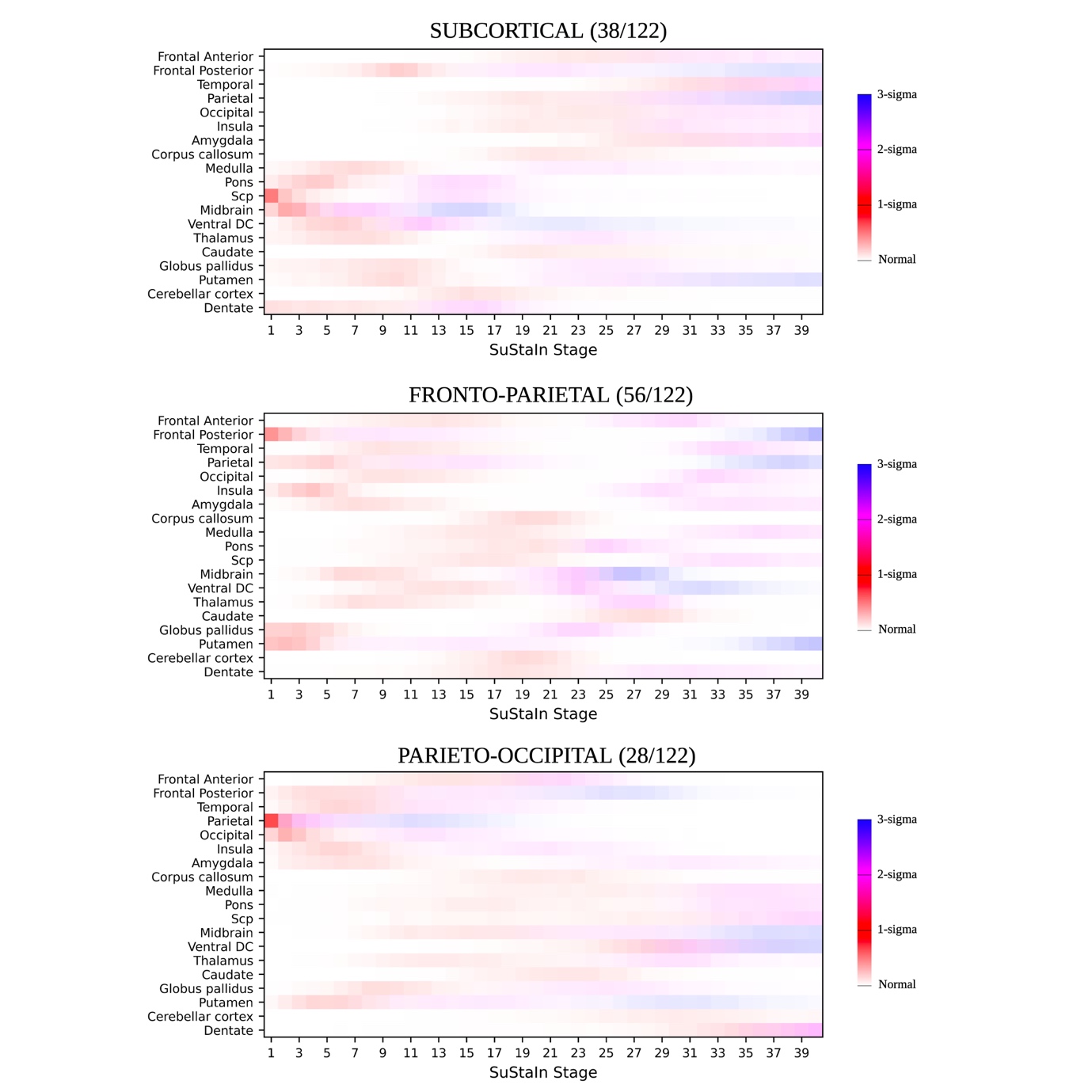
**

**Supplementary Figure 7 – Positional variance diagrams for SuStaIn subtypes in the 3-subtype model.** These represent the uncertainty in the subtype progression patterns for each region. Each region (y-axis) is shaded based on the probability a particular z score is reached at a particular SuStaIn stage (x-axis). Z scores range from zero (white), one (red), two (pink) to three (blue) as shown in the bar on the right hand side of figure. Total subtypable baseline scans =122 (*Subcortical* 38, *Fronto-parietal* 56, *Parieto-occipital* 28).

**
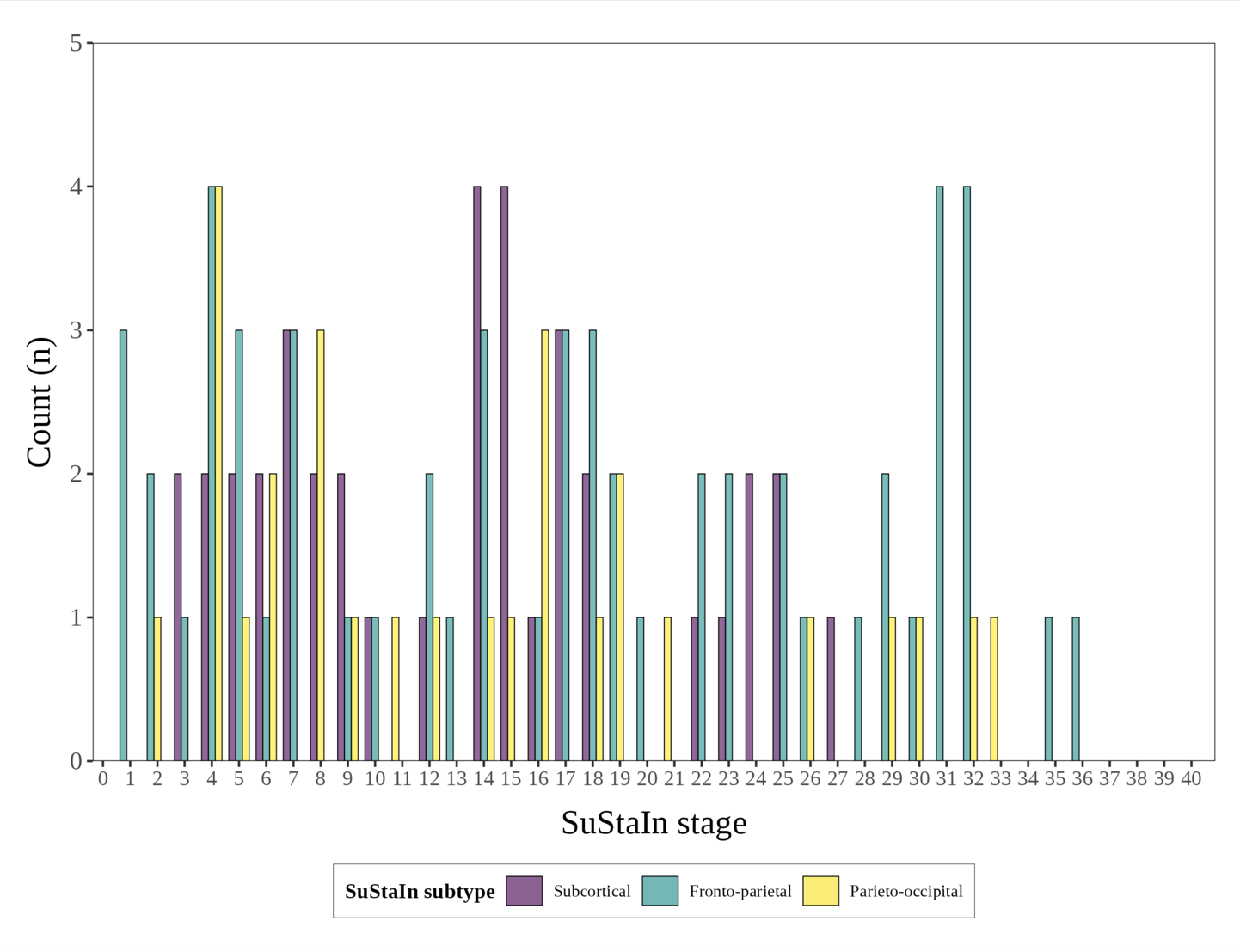
**

**Supplementary Figure 8 – Stage distribution by Subtype for the 3-subtype model.** Individual bars represent number (n) of cases estimated to be a particular stage by the SuStaIn algorithm, with the colour of the bar representing the subtype those cases were assigned to; purple = *Subcortical*, green = *Fronto-parietal*, yellow = *Parieto-occipital*.


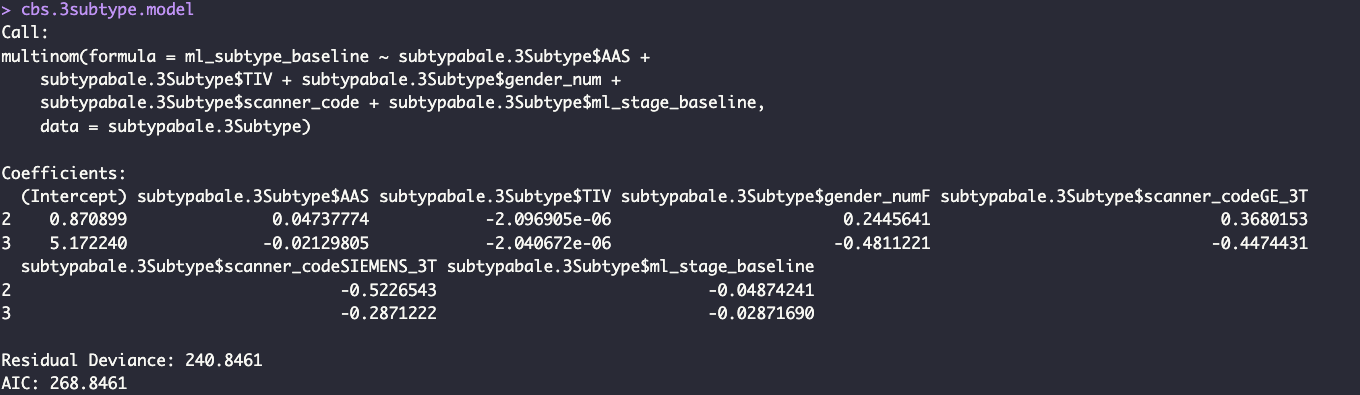

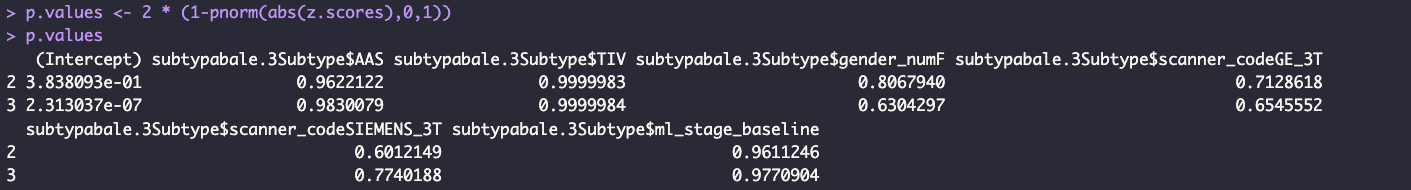


**Supplementary Figure 9 – Three-subtype multinomial logistic regression model output.** To test for any residual association between covariates (scanner magnetic field strength, scanner manufacturer, sex, AAS, TIV, SuStaIn stage) and SuStaIn subtype, by fitting a multinomial logistic regression model to the three-subtype data. Analysis was performed using the *multinom*() function from the nnet package (version 7.3-19) in R version 4.0.5. Abbreviations: TIV = total intracranial volume, AAS = age at scan.

### **Supplementary Tables**

Supplementary Table 1 - GIF subregions included in each cortical and cerebellar region used as SuStaIn input.

| Regions included in SuStaIn | GIF Subregions |
| --- | --- |
| Frontal Anterior | Frontal operculum, central operculum, frontal pole, gyrus rectus, middle frontal cortex, subcallosal area, superior frontal gyrus medial segment, superior frontal gyrus, middle frontal gyrus, opercular part of the inferior frontal gyrus, orbital part of the inferior frontal gyrus, triangular part of the inferior frontal gyrus, anterior orbital gyrus, medial orbital gyrus, lateral orbital gyrus, posterior orbital gyrus |
| Frontal Posterior | Precentral gyrus, precentral gyrus medial segment, supplementary motor cortex |
| Temporal | Entorhinal area, fusiform gyrus, parahippocampal gyrus, inferior temporal gyrus, middle temporal gyrus, superior temporal gyrus, temporal pole, planum polare, planum temporale, transverse temporal gyrus |
| Parietal | Precuenus, parietal operculum, supramarginal gyrus, superior parietal lobule, angular gyrus, postcentral gyrus, postcentral gyrus medial segment |
| Occipital | Cuneus, calcarine cortex, lingual gyrus, occipital fusiform gyrus, superior occipital gyrus, inferior occipital gyrus, middle occipital gyrus, occipital pole |
| Insula | Anterior insula, posterior insula |
| Amygdala | Amygdala |
| Corpus Callosum | Corpus Callosum |
| Medulla | Medulla |
| Pons | Pons |
| Superior Cerebellar Peduncles | Superior cerebellar peduncles |
| Midbrain | Midbrain |
| Ventral Diencephalon | Ventral Diencephalon (GIF segmentation includes subthalamic nucleus, substantia nigra and hypothalamus) |
| Thalamus | Thalamus |
| Caudate | Caudate |
| Globus Pallidus | Globus Pallidus |
| Putamen | Putamen |
| Cerebellar Cortex | Lobules I/IV, V, VI, VIIA-Crus I, VIIA-Crus II, VIIB, VIIIA, VIIB, IX, X |
| Dentate | Dentate nucleus |

Supplementary Table 2 - Effect size (Cohen's $\boldsymbol{d}$) by region of interest.

| Region of Interest | Cohen’s $\boldsymbol{d}$ |
| --- | --- |
| Putamen | 1.62 |
| Frontal Posterior | 1.49 |
| Midbrain | 1.44 |
| Thalamus | 1.43 |
| Parietal | 1.37 |
| Insula | 1.36 |
| Globus pallidus | 1.22 |
| Pons | 1.13 |
| Amygdala | 1.07 |
| Ventral DC | 1.06 |
| SCP | 1.05 |
| Temporal | 0.90 |
| Occipital | 0.89 |
| Frontal Anterior | 0.88 |
| Caudate | 0.85 |
| Dentate | 0.78 |
| Corpus callosum | 0.73 |
| Medulla | 0.72 |
| Cerebellar cortex | 0.66 |
| NA | 0.59^a^ |
| Basal forebrain | 0.59^a^ |
| Cingulate | 0.50^a^ |
| Vermis | 0.49^a^ |
| Hippocampus | 0.45^a^ |

Cohen’s $d$ calculated as the standardised mean difference between adjusted the regional volume of that that region of interest (ROI) in cases vs controls. ^a^ a threshold of greater than or equal to 0.6 was used to select ROI as input for SuStaIn algorithm resulting in 19 biomarkers being included in model. Abbreviations: DC = diencephalon, SCP = superior cerebellar peduncles, NA = nucleus accumbens

Supplementary Table 3 - SuStaIn algorithm settings for each biomarker

| Biomarker | R | $\boldsymbol{Z}_{\boldsymbol{max}}$ |
| --- | --- | --- |
| Frontal Anterior | 2 | 5 |
| Frontal Posterior | 3 | 5 |
| Temporal | 2 | 5 |
| Parietal | 3 | 6 |
| Occipital | 2 | 5 |
| Insula | 2 | 5 |
| Amygdala | 2 | 4 |
| Corpus Callosum | 1 | 3 |
| Medulla | 2 | 4 |
| Pons | 2 | 4 |
| SCP | 2 | 4 |
| Midbrain | 3 | 6 |
| Ventral DC | 3 | 5 |
| Thalamus | 2 | 5 |
| Caudate | 1 | 4 |
| GP | 2 | 4 |
| Putamen | 3 | 5 |
| Cerebellar cortex | 1 | 3 |
| Dentate | 2 | 4 |

R is the number of z-scores included for biomarker $i$, and $Z_{max}$ is maximum z-score modelled for biomarker $i$. Total number of biomarkers $\left( i \right)$ for model = 40. C_max_ (the maximum number of subtypes fitted) = 3. Model uncertainty was estimated using 100,000 Markov Chain Monte Carlo (MCMC) iterations. In the single-cluster expectation maximisation procedure the single-cluster sequence was optimised from 24 different random starting sequences to find the maximum likelihood solution.

**Supplementary Table 4: Linear models of covariate adjusted regional volumes by age of scan (Cases)**

| **Region of Interest** | **Estimate** | **Standard Error** | $\boldsymbol{t}$ **value** | $\boldsymbol{R}^{\boldsymbol{2}}$ | $\boldsymbol{p}$ **value^a^** |
| --- | --- | --- | --- | --- | --- |
| Frontal Anterior | 111.1 | 150.3 | 0.74 | -0.003 | 1.00 |
| Frontal Posterior | -26.4 | 50.7 | -0.52 | -0.005 | 1.00 |
| Temporal | 179.5 | 125.1 | 1.43 | 0.008 | 1.00 |
| Parietal | 266.3 | 122.4 | 2.18 | 0.027 | 0.60 |
| Occipital | 41.7 | 89.2 | 0.47 | -0.006 | 1.00 |
| Insula | 10.1 | 13.1 | 0.77 | -0.003 | 1.00 |
| Amygdala | 0.6 | 3.8 | 0.16 | -0.007 | 1.00 |
| Corpus callosum | 29.5 | 24.8 | 1.19 | 0.003 | 1.00 |
| Medulla | -12.1 | 6.4 | -1.88 | 0.019 | 1.00 |
| Pons | -35.4 | 19.6 | -1.8 | 0.017 | 1.00 |
| Scp | -1.1 | 0.5 | -2.11 | 0.025 | 0.70 |
| Midbrain | -17.8 | 8.7 | -2.05 | 0.023 | 0.81 |
| Ventral DC | -19.4 | 9.6 | -2.03 | 0.023 | 0.84 |
| Thalamus | 3.5 | 10.3 | 0.34 | -0.007 | 1.00 |
| Caudate | 15 | 9.5 | 1.58 | 0.011 | 1.00 |
| GP | -0.6 | 3.7 | -0.15 | -0.007 | 1.00 |
| Putamen | 3.5 | 8.4 | 0.42 | -0.006 | 1.00 |
| Cerebellar cortex | -118.7 | 94.2 | -1.26 | 0.004 | 1.00 |
| Dentate | -13.1 | 6.2 | -2.41 | 0.061 | 0.06 |

A linear model was fit to each region of interest covariate adjusted volumes (ROI ~ age at scan).

^a^ $p$ values were Bonferroni corrected for multiple comparisons

Abbreviations: DC = diencephalon, GP = globus pallidus, NA = nucleus accumbens, ROI = region of interest

**Supplementary Table 5: Linear models of covariate adjusted regional volumes by age of scan (Controls)**

| **Region of Interest** | **Estimate** | **Standard Error** | $\boldsymbol{t}$ **value** | $\boldsymbol{R}^{\boldsymbol{2}}$ | $\boldsymbol{p}$ **value^a^** |
| --- | --- | --- | --- | --- | --- |
| Frontal Anterior | 2.07E-09 | 73.6 | 2.82E-11 | -0.004 | 1.00 |
| Frontal Posterior | 4.29E-10 | 24.9 | 1.72E-11 | -0.004 | 1.00 |
| Temporal | -6.89E-09 | 55.9 | -1.23E-10 | -0.004 | 1.00 |
| Parietal | -1.35E-08 | 48.9 | -2.76E-10 | -0.004 | 1.00 |
| Occipital | -3.00E-09 | 40.2 | -7.47E-11 | -0.004 | 1.00 |
| Insula | -1.92E-09 | 6.7 | -2.86E-10 | -0.004 | 1.00 |
| Amygdala | -8.08E-10 | 2.0 | -4.03E-10 | -0.004 | 1.00 |
| Corpus callosum | -5.56E-09 | 14.9 | -3.73E-10 | -0.004 | 1.00 |
| Medulla | -8.20E-10 | 3.6 | -2.27E-10 | -0.004 | 1.00 |
| Pons | -2.34E-09 | 10.6 | -2.22E-10 | -0.004 | 1.00 |
| Scp | -3.27E-11 | 0.3 | -1.17E-10 | -0.004 | 1.00 |
| Midbrain | -9.00E-10 | 3.4 | -2.62E-10 | -0.004 | 1.00 |
| Ventral DC | -1.34E-09 | 4.0 | -3.39E-10 | -0.004 | 1.00 |
| Thalamus | -1.24E-09 | 5.5 | -2.25E-10 | -0.004 | 1.00 |
| Caudate | 3.22E-10 | 5.5 | 5.86E-11 | -0.004 | 1.00 |
| GP | -3.45E-10 | 2.2 | -1.59E-10 | -0.004 | 1.00 |
| Putamen | -1.11E-09 | 4.7 | -2.36E-10 | -0.004 | 1.00 |
| Cerebellar cortex | -1.08E-08 | 50.9 | -2.13E-10 | -0.004 | 1.00 |
| Dentate | 2.83E-10 | 2.2 | 1.29E-10 | -0.004 | 1.00 |

A linear model was fit to each region of interest covariate adjusted volumes (ROI ~ age at scan).

^a^ $p$ values were Bonferroni corrected for multiple comparisons

Abbreviations: DC = diencephalon, GP = globus pallidus, NA = nucleus accumbens, ROI = region of interest

Supplementary Table 6 - Longitudinal consistency of subtype assignments for two-subtype model.

|  | Classification follow-up visit | | |
| --- | --- | --- | --- |
| Classification previous visit | Normal appearing^a^ | Subcortical subtype | Fronto-parieto-occipital subtype |
| **Normal appearing^a^** | 10 (91%) | 0 (0%) | **1 (9%)^b^** |
| **Subcortical subtype** | 0 (0%) | **36 (100%)^b^** | 0 (0%) |
| **Fronto-parieto-occipital subtype** | 0 (0%) | 2 (3%) | **64 (97%)^b^** |

^a^ Normal appearing = not subtypable (Stage 0). Note that this only includes 11 individuals that were not subtypable at baseline and had a follow-up scan. An observation is longitudinally consistent (^b^) if individuals remain in the same group or progress from the normal-appearing group to a SuStaIn subtype at follow-up visit. Entries indicate the number of visits n, with the % of the total individuals in classification at previous visit in classification at follow-up in brackets. Longitudinally consistent observations highlighted in bold.

Supplementary Table 7 - Longitudinal consistency of subtype assignments for three subtype model

|  | Classification follow-up visit | | | |
| --- | --- | --- | --- | --- |
| Classification previous visit | Normal appearing^a^ | Subcortical subtype | Fronto-parietal subtype | Parieto-occipital subtype |
| **Normal appearing^a^** | 10 (91%) | 0 (0%) | 0 (0%) | **1 (9%)^b^** |
| **Subcortical subtype** | 0 (0%) | **21 (80%)^b^** | 5 (20%) | 0 (0%) |
| **Fronto-parietal subtype** | 0 (0%) | 0 (0%) | **53 (96%)^b^** | 2 (4%) |
| **Parieto-occipital subtype** | 0 (0%) | 0 (0%) | 0 (0%) | **21 (100%)^b^** |

^a^ Normal appearing = not subtypable (Stage 0). Note that this only includes 11 individuals that were not subtypable at baseline and had a follow-up scan. An observation is longitudinally consistent (^b^) if individuals remain in the same group or progress from the normal-appearing group toa SuStaIn subtype at follow-up. Entries indicate the number of visits n, with the % of the total individuals in classification at previous visit in classification at follow-up in brackets. Longitudinally consistent observations highlighted in bold.

Supplementary Table 8 - Regional brain volumes by subtype in the two-subtype model

| Region | Subcortical | Fronto-parieto-occipital | $\boldsymbol{p}$ value |
| --- | --- | --- | --- |
| Frontal Anterior | 128935 (13578) | 126452 (16259) | 0.36 |
| Frontal Posterior | 34150 (4371) | 32601 (4930) | 0.07 |
| Temporal | 117607 (10436) | 109989 (13026) | 4.6 x 10^-4a^ |
| Parietal | 85183 (9049) | 76286 (10901) | 2.5 x 10^-6a^ |
| Occipital | 68530 (7469) | 63927 (8536) | 0.02 |
| Cingulate | 26734 (2915) | 26268 (3409) | 0.41 |
| Insula | 9525 (1255) | 9193 (1316) | 0.16 |
| Amygdala | 3301 (339) | 3114 (372) | 4.3 x 10^-3a^ |
| Corpus callosum | 18084 (2796) | 17992 (2673) | 0.85 |
| Medulla | 4597 (646) | 4809 (590) | 0.06 |
| Pons | 12703 (1634) | 13984 (1600) | 2.7 x 10^-5a^ |
| SCP | 196 (45) | 230 (47) | 5.4 x 10^-5a^ |
| Midbrain | 5172 (759) | 5778 (703) | 1.3 x 10^-5a^ |
| Ventral DC | 7837 (857) | 8438 (973) | 3.9 x 10^-4a^ |
| Thalamus | 9727 (1002) | 10110 (926) | 0.03 |
| Caudate | 6248 (821) | 6177 (901) | 0.65 |
| GP | 3367 (349) | 3383 (373) | 0.81 |
| Putamen | 7759 (726) | 7557 (811) | 0.15 |
| Cerebellar_cortex | 86575 (8384) | 88222 (8993) | 0.30 |
| Dentate | 2699 (376) | 3023 (399) | 9.4 x 10^-6a^ |
| Hippocampus | 7361 (768) | 7187 (662) | 0.19 |
| Vermis | 4359 (532) | 4418 (491) | 0.52 |
| NA | 1051 (111) | 1042 (126) | 0.68 |
| Basal forebrain | 982 (98) | 975 (106) | 0.73 |

Values are mean volume (mm^3^) for that brain region (SD). Two-tailed, unpaired $t$ tests performed. ^a^statistically significant at p < 0.05, corrected for multiple comparisons (Bonferroni). Abbreviations: SCP = superior cerebellar peduncles, DC = diencephalon, GP = globus pallidus, NA = nucleus accumbens

Supplementary Table 9 - Regional brain volumes by subtype in the three-subtype model

| Region | Subcortical | Fronto-parietal | Parieto-occipital | $\boldsymbol{p}$ value |
| --- | --- | --- | --- | --- |
| Frontal Anterior | 131521 (11495) | 124941 (14110) | 127361 (20123) | 0.12 |
| Frontal Posterior | 34650 (4185) | 32087 (3831) | 33841 (6407) | 0.03 |
| Temporal | 119008 (9222) | 112067 (10712) | 108583 (16717) | 1.6 x 10^-3a^ |
| Parietal | 87136 (7631) | 79395 (8404) | 72733 (13935) | 1.4 x 10^-7a^ |
| Occipital | 69087 (7654) | 66254 (7431) | 61235 (9222) | 5.8 x 10^-4a^ |
| Cingulate | 27080 (2484) | 26101 (3154) | 26437 (4053) | 0.35 |
| Insula | 9807 (1109) | 9129 (1192) | 9136 (1594) | 0.03 |
| Amygdala | 3358 (272) | 3112 (337) | 3156 (474) | 4.4 x 10^-3a^ |
| Corpus callosum | 18127 (2414) | 17642 (2575) | 18774 (3287) | 0.20 |
| Medulla | 4507 (644) | 4794 (616) | 4820 (570) | 0.051 |
| Pons | 12513 (1471) | 13700 (1834) | 13985 (1435) | 4.3 x 10^-4a^ |
| SCP | 182 (40) | 225 (47) | 237 (43) | 1.2 x 10^-6a^ |
| Midbrain | 5074 (756) | 5596 (720) | 5894 (722) | 1.4 x 10^-5a^ |
| Ventral DC | 7738 (845) | 8116 (852) | 8843 (1010) | 1.1 x 10^-5a^ |
| Thalamus | 9686 (932) | 9900 (979) | 10342 (950) | 0.02 |
| Caudate | 6244 (740) | 6123 (862) | 6337 (1031) | 0.56 |
| GP | 3414 (323) | 3292 (352) | 3491 (403) | 0.043 |
| Putamen | 7893 (681) | 7449 (722) | 7723 (926) | 0.020 |
| Cerebellar cortex | 86043 (7531) | 86829 (9419) | 90988 (8156) | 0.05 |
| Dentate | 2652 (416) | 2893 (355) | 3142 (397) | 6.8 x 10^-6a^ |
| Vermis | 4378 (500) | 4330 (543) | 4540 (439) | 0.20 |
| Hippocampus | 7579 (686) | 7186 (728) | 7003 (597) | 2.3 x 10^-3a^ |
| NA | 1068 (99) | 1016 (116) | 1076 (141) | 0.04 |
| Basal forebrain | 981 (93) | 978 (93) | 979 (132) | 0.99 |

Values are mean volume (mm^3^) for that brain region (SD). Group comparisons were performed using a linear model for continuous variables (continuous variable ~ SuStaIn subtype). ^a^statistically significant at p < 0.05, corrected for multiple comparisons (Bonferroni). Abbreviations: SCP = superior cerebellar peduncles, DC = diencephalon, GP = globus pallidus, NA = nucleus accumbens

### **Bibliography**

1. Young AL, Marinescu R V., Oxtoby NP, et al. Uncovering the heterogeneity and temporal complexity of neurodegenerative diseases with Subtype and Stage Inference. *Nat Commun*. 2018;9(1). doi:10.1038/s41467-018-05892-0

2. Kass R, Raferty A. Bayes Factors. *Journal of the American Statistical Association*. 1995;90(430):773-795.

3. Bates D, Mächler M, Bolker B, Walker S. Fitting Linear Mixed-Effects Models Using lme4. *Journal of Statistical Software*. 2015;67(1):1-48. doi:10.18637/JSS.V067.I01
